# The Sequence-Specific Transcription Factor c-Jun Targets Cockayne Syndrome Protein B to Regulate Transcription and Chromatin Structure

**DOI:** 10.1371/journal.pgen.1004284

**Published:** 2014-04-17

**Authors:** Robert J. Lake, Erica L. Boetefuer, Pei-Fang Tsai, Jieun Jeong, Inchan Choi, Kyoung-Jae Won, Hua-Ying Fan

**Affiliations:** 1 Epigenetics Program, Perelman School of Medicine, University of Pennsylvania, Philadelphia, Pennsylvania, United States of America; 2 Department of Biochemistry and Biophysics, Perelman School of Medicine, University of Pennsylvania, Philadelphia, Pennsylvania, United States of America; 3 Biology Graduate Program, Graduate School of Arts and Sciences, University of Pennsylvania, Philadelphia, Pennsylvania, United States of America; 4 Institute for Diabetes Obesity and Metabolism, Perelman School of Medicine, University of Pennsylvania, Philadelphia, Pennsylvania, United States of America; 5 Department of Genetics, Perelman School of Medicine, University of Pennsylvania, Philadelphia, Pennsylvania, United States of America; University of Washington, United States of America

## Abstract

Cockayne syndrome is an inherited premature aging disease associated with numerous developmental and neurological defects, and mutations in the gene encoding the CSB protein account for the majority of Cockayne syndrome cases. Accumulating evidence suggests that CSB functions in transcription regulation, in addition to its roles in DNA repair, and those defects in this transcriptional activity might contribute to the clinical features of Cockayne syndrome. Transcription profiling studies have so far uncovered CSB-dependent effects on gene expression; however, the direct targets of CSB's transcriptional activity remain largely unknown. In this paper, we report the first comprehensive analysis of CSB genomic occupancy during replicative cell growth. We found that CSB occupancy sites display a high correlation to regions with epigenetic features of promoters and enhancers. Furthermore, we found that CSB occupancy is enriched at sites containing the TPA-response element. Consistent with this binding site preference, we show that CSB and the transcription factor c-Jun can be found in the same protein-DNA complex, suggesting that c-Jun can target CSB to specific genomic regions. In support of this notion, we observed decreased CSB occupancy of TPA-response elements when c-Jun levels were diminished. By modulating CSB abundance, we found that CSB can influence the expression of nearby genes and impact nucleosome positioning in the vicinity of its binding site. These results indicate that CSB can be targeted to specific genomic loci by sequence-specific transcription factors to regulate transcription and local chromatin structure. Additionally, comparison of CSB occupancy sites with the MSigDB Pathways database suggests that CSB might function in peroxisome proliferation, EGF receptor transactivation, G protein signaling and NF-κB activation, shedding new light on the possible causes and mechanisms of Cockayne syndrome.

## Author Summary

Cockayne syndrome is a devastating inherited disease, in which patients appear to age prematurely, have sun sensitivity and suffer from profound neurological and developmental defects. Mutations in the CSB gene account for the majority of Cockayne syndrome cases. CSB is an ATP-dependent chromatin remodeler, and these proteins can use energy from ATP-hydrolysis to alter contacts between DNA and histones of a nucleosome, the basic units of chromatin structure. CSB functions in DNA repair, but accumulating evidence reveals that CSB also functions in transcription regulation. Here, we determined the genomic localization of CSB to identify its gene targets and found that CSB occupancy displays high correlation to regions with epigenetic features of promoters and enhancers. Furthermore, CSB is enriched at genomic regions containing the binding site for the c-Jun transcription factor, and we found that these two proteins interact, uncovering a new targeting mechanism for CSB. We also demonstrate that CSB can influence gene expression in the vicinity of its binding sites and alter local chromatin structure. Together, this study supports the hypothesis that defects in the regulation of gene expression and chromatin structure by CSB might contribute to the diverse clinical features of Cockayne syndrome.

## Introduction

Cockayne syndrome is a devastating inherited disease in which patients have features of premature aging, display increased sun sensitivity, and suffer from profound neurological and developmental defects [1]. Mutations in the gene encoding the CSB (Cockayne syndrome complementation B) protein are associated with the majority of Cockayne syndrome cases. CSB belongs to the SWI2/SNF2 ATP-dependent chromatin remodeling protein family [2]. ATP-dependent chromatin remodelers are conserved from yeast to human, and they are critical in regulating fundamental nuclear processes, such as transcription and DNA repair [3], [4]. These proteins use ATP as energy to alter chromatin structure non-covalently, resulting in changes in nucleosome position, composition or conformation. By doing so, ATP-dependent chromatin remodelers can regulate the access of protein factors to DNA. Additionally, some ATP-dependent chromatin remodelers can assemble nucleosomes or create equally spaced nucleosomes to facilitate the formation of higher-order chromatin structure [5]. Most remodelers in isolation can alter chromatin structure *in vitro*. The additional proteins that form complexes with ATP-dependent chromatin remodelers are often involved in enhancing the specific activity of the remodeler or targeting the remodeling complex to specific genomic regions [6]–[8]. Additionally, some SWI2/SNF2 family members can alter contacts between DNA and non-histone proteins [9]. For example, the MOT1 remodeler can use the energy from ATP hydrolysis to dissociate TBP (TATA box binding protein) from DNA [9].

*In vitro*, the CSB remodeler appears to interact with DNA in a sequence-independent manner, and CSB displays both DNA and nucleosome stimulated ATP hydrolysis activity [4], [10]–[12]. The biochemical activities that have been associated with CSB are quite diverse [2]. For example, CSB has been shown to alter DNA conformation and to actively wrap DNA [13], [14], both in an ATP-dependent manner. Additionally, CSB has been shown to use the energy from ATP hydrolysis to alter chromatin structure [8], [13]. Most recently, the NAP1-like histone chaperone was shown to significantly enhance the chromatin remodeling activity of CSB and, together, these two proteins can centralize mononucleosomes, suggesting a potential function of CSB in nucleosome spacing [8], [15], [16]. Several ATP-independent activities have also been ascribed to CSB. Such activities include dissociating non-histone proteins from DNA, annealing single-stranded DNA, and modulating the activities of DNA repair proteins [17].

CSB is best known for its function in transcription-coupled DNA repair, a process that preferentially removes bulky DNA lesions that stall transcription, such as those created by UV irradiation [18], [19]. CSB is one of the first proteins recruited to sites of lesion-stalled transcription, and the ability of CSB to hydrolyze ATP is essential for this association [20]. After its arrival to lesion-stalled transcription, CSB appears to have both chromatin remodeling-dependent and remodeling-independent functions. One function of CSB is to recruit the DNA repair machinery [20], and this activity does not rely upon nucleosome repositioning by CSB [8]. The chromatin remodeling activity of CSB, on the other hand, is likely to be important for regulating protein DNA associations or nucleosome positioning necessary for the repair process or the resumption of transcription after repair. CSB is also involved in the repair of oxidative lesions in both nuclear and mitochondrial DNA [21]–[24].

In addition to its well-documented function in DNA repair, accumulating evidence indicates that CSB also participates in transcription regulation [25]–[28]. CSB has been found to be in complexes with both RNA polymerase II and RNA polymerase I. Interestingly, CSB does not appear to impact transcription in a general manner, as transcription-profiling experiments revealed that CSB has specific effects on gene expression. The results of that study has lead to the intriguing hypothesis that Cockayne syndrome might be, at least in part, a disease of transcription deregulation [28], [29].

To understand better how CSB carries out its diverse functions and to gain potential insights into the underlying mechanism of Cockayne syndrome, we performed a genome-wide study of CSB occupancy to elucidate the mechanisms of CSB targeting and to understand the impacts of CSB occupancy on transcription regulation.

## Results

### The genomic occupancy of CSB

**Figure 1:**
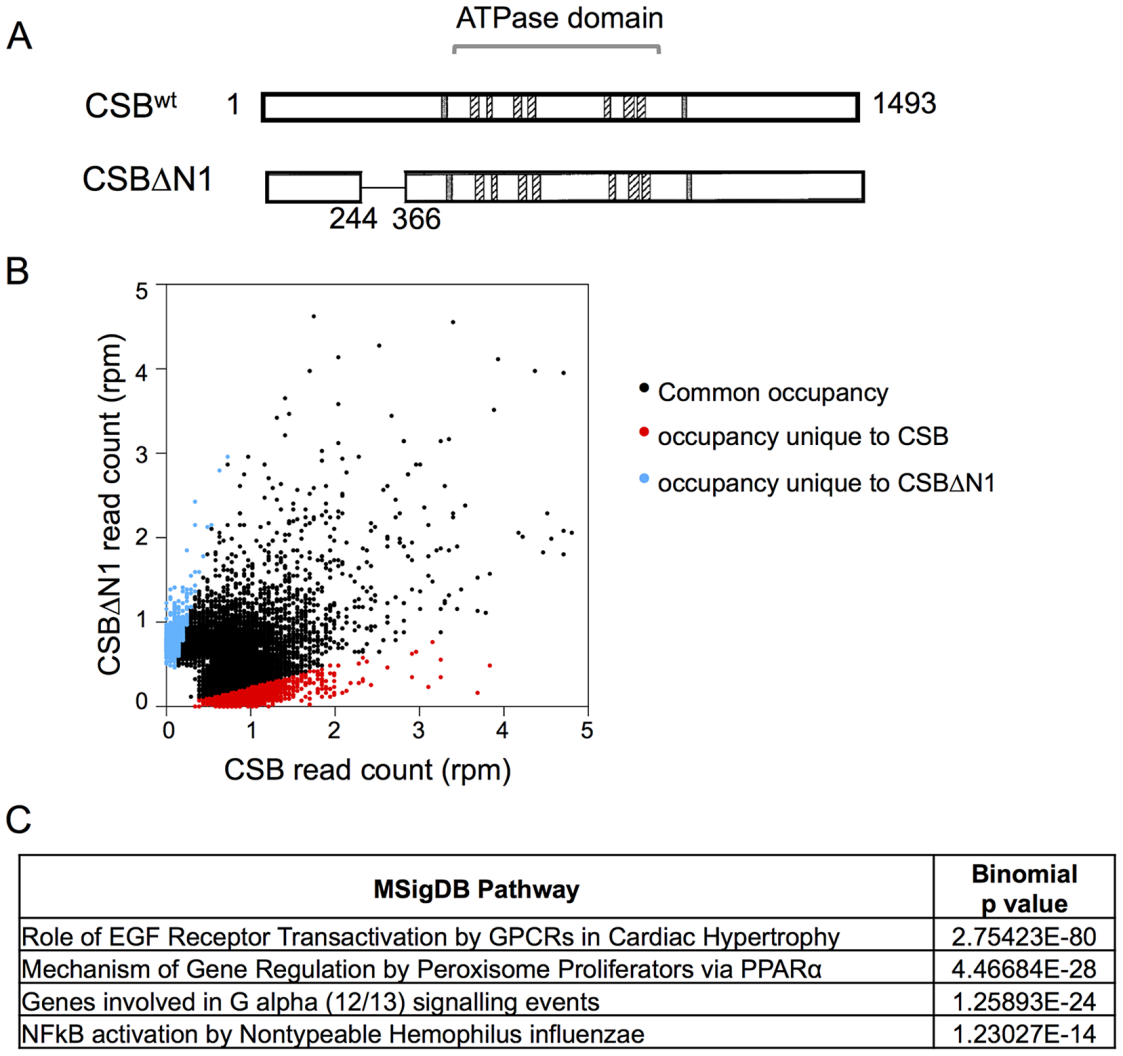
Overview of CSB and CSBΔN1 ChIP-seq data. (A) Schematics of CSB and CSBΔN1 proteins. The central ATPase domain consists of seven conserved helicase motifs (striped boxes) and is flanked by two putative nuclear localization sequences (gray). (B) Scatter plot showing the correlation between CSB and CSBΔN1 ChIP-seq results: common occupancy sites (black), peaks unique to CSB (red) and peaks unique to CSBΔN1 (blue). rpm is reads per million. (C) MSigDB pathways of peaks common to CSB and CSBΔN1.

To identify genomic regions that CSB occupies, we performed chromatin immunoprecipitation using a monoclonal antibody raised against CSB, followed by deep sequencing. CS1AN-sv cells that were reconstituted with CSB were used for these experiments, as CS1AN-sv cells do not express the alternatively spliced CSB-PiggyBac fusion protein (Figure S1) [30]; this cell line is hemizygous for the CSB locus and the retained CSB allele has a premature stop codon at amino acid 337 [19] (see Materials and Methods). Additionally, to understand the importance of CSB's nucleosome-remodeling activity in CSB function, we also included in these assays the remodeling-defective CSBΔN1 derivative (Figure 1A) [8].

The resulting sequencing reads were mapped to the human genome (HG19 assembly) using the Bowtie aligner [31]. Peaks were identified using HOMER (Hypergeometric Optimization of Motif EnRichment) with a default option (FDR = 0.001 and Poisson p-value cut-off = 0.0001) on ChIPed samples against matching input samples. In total, we recovered 17,779 CSB peaks and 3,607 CSBΔN1 peaks (Table 1).

**Table 1:** Summary of sequence reads from CSB and CSBΔN1 ChIP-seq.

ChIP-seq	Total reads	Mapped reads	Unique reads	Total peaks	Unique peaks	Common peaks
CSB	26,707,065	20,511,328	14,569,874	17,779	6,398 (36%)	11,381 (64%)
CSBΔN1	63,244,095	43,077,476	34,183,773	3,607	877 (24%)	2,730 (76%)

We subsequently classified peaks unique to CSB or CSBΔN1 (Figure 1B). To do this, we compared signal intensities for each CSB peak identified by HOMER to the CSBΔN1 signal intensity, regardless of whether the CSBΔN1 signal was identified as a peak by HOMER. Similarly, we compared signal intensities for each CSBΔN1 peak identified by HOMER to the CSB signal intensity, regardless of whether the CSB signal was identified as a peak by HOMER. Signal intensities were compared over a 200 bp region. If the difference between signal intensities was 4-fold or greater and the p-value for that difference was ≤0.0001, the signal was classified as unique; the remaining signals were classified as common (Tables S1, S2). Among them, we identified 6,398 peaks unique to CSB and 877 peaks unique to CSBΔN1 (Table 1). As shown in Figure 1B, ∼36% of the CSB occupancy sites were unique to CSB and ∼24% of the CSBΔN1 occupancy sites were unique to CSBΔN1.

We then classified the CSB and CSBΔN1 occupancy sites into seven functional categories (see Materials and Methods), using the UCSC RefSeq gene annotations and the CEAS package [32]. The distribution of CSB peaks among those categories was largely consistent with a random distribution (Figure S2); however, we observed modest enrichment of CSB occupancy at promoter regions (1.5% for CSB peaks vs. 1.1% for the genomic distribution, binomial test p-value of 7.3e-07) and modest depletion at 3′UTRs (1.1% for CSB peaks vs. 1.4% for the genomic distribution, binomial test p-value of 1.6e-05). Interestingly, CSBΔN1 peaks displayed greater enrichment at promoter regions (3.1% for CSBΔN1 peaks vs. 1.1% for the genomic distribution, binomial test p-value of 6.8e-23) and 5′UTRs (1.1% for CSB peaks vs. 0.4% for the genomic distribution, binomial test p-value of 1.4e-09) (Figure S2).

Given that CSBΔN1 is more significantly enriched at promoters and 5′ UTRs than CSB (p-values of 5.2e-11 and 1.8e-7, respectively) (Figure S2), we calculated the number of CSB and CSBΔN1 peaks as a function of distance from transcription start sites (TSS) and plotted the results as a histogram using a bin size of 500 bp. As shown in Figure S3, CSB displayed only a modest increase in occupancy around TSSs, while the remodeling-deficient CSBΔN1 derivative displayed greater occupancy around TSSs. These results are consistent with our functional genomic distribution analysis, which indicated the CSBΔN1 displayed a more significant enrichment at promoter regions (Figure S2).

We next classified the genomic localization of the common and unique occupancy sites into the seven functional categories described above (Figure S4). We found that the common CSB and CSBΔN1 occupancy sites were over-represented at promoters and 5′UTRs as compared to the genomic distribution (2% vs 1.1%, p-value of 4.3e-20 and 0.7% vs 0.3%, p-value of 6.1e-8, respectively). The occupancy sites unique to CSBΔN1 were slightly over-represented at promoter regions (3% vs 1.1%, p-value of 2e-06), while the occupancy sites unique to CSB were similar to the genomic distribution.

### CSB is enriched at sites containing promoter and enhancer features

To gain further insight into the potential functions of CSB, we classified CSB and CSBΔN1 occupancy sites according to the 15 chromatin states defined by Ernst et al. (2011), which are largely based on the presence or absence of specific histone marks (Table 2) [33], [34]. For this analysis, we used the classifications from normal lung fibroblasts, as CS1AN-sv cells are also a fibroblast cell line and, therefore, these two lines are most similar [34]. In agreement with the functional classification described above, CSB and CSBΔN1 displayed significant enrichment at transcribing promoters (Table 2): 2.7% of the CSB peaks (p-value of 3.2e-58) and 7.3% of the CSBΔN1 peaks (p-value of 4.1e-119) occupied active and weak promoters. Strikingly, sites of CSB and CSBΔN1 occupancy displayed a strong correlation with strong enhancers (Table 2). Moreover, while regions containing the H3K4me1 (a mark often associated with enhancers) represented only 4.4% of fibroblast chromatin, this histone mark was present at ∼19% of the CSB occupancy sites (p-value of 7.9e-1141) and ∼26% of CSBΔN1 occupancy sites (p-value of 1.7e-412). Moreover, ∼29% of the top CSB peaks were associated with enhancer features (p-value of 1.2e-486). Taken together, these results are consistent with the notion that CSB is involved in the regulation of gene expression [25]–[28].

**Table 2:** Classification of CSB and CSBΔN1 peaks according to chromatin features defined in Ernst et al. (2011).

chromatin features			all CSB peaks			top CSB peaks^1^			all CSBΔN1 peaks		
chromatin state	enriched chromatin marks^2^	% coverage	% coverage	fold^3^	p-value	% coverage	fold^3^	p-value	% coverage	fold^3^	p-value
1. Active Promoter	H3K4me2-3, H3K27Ac, H3K9Ac	0.6	1.6	2.5	1.9e-30	2.5	4.0	5.1e-26	4.6	7.3	8.4e-87
2. Weak Promoter	H3K4me2-3	0.5	1.1	2.1	7.3e-21	1.8	3.4	4.2e-15	2.6	4.9	5.2e-35
Transcribing promoter (1,2)	H3K4me2-3	1.1	2.7	2.3	3.2e-58	4.3	3.8	1.8e-39	7.3	6.3	4.1e-119
3. Poised Promoter	H3K27me3, H3K4me2	0.1	0.2	1.4	3.1e-02	0.2	1.1	4.8e-1	0.3	2.4	5.5e-3
4. Strong Enhancer	H3K4me1-3, H3K27Ac, H3K9Ac	0.3	2.2	6.4	1.1e-171	3.2	9.4	2.3e-64	3.1	9.2	2.9e-69
5. Strong Enhancer	H3K4me1, H3K27Ac	1.3	8.8	7.0	2.1e-769	15.7	12.4	8.5e-381	12.4	9.8	3.4e-287
6. Weak Enhancer	H3K4me1, H3K27me2	0.8	2.5	2.9	9.2e-84	3.1	3.6	1.4e-27	3.5	4.2	2.8e-41
7. Weak Enhancer	H3K4me1	1.9	5.6	2.9	3.8e-191	6.9	3.6	6.7e-60	6.0	3.1	8.8e-48
all enhancer (4–7)	H3K4me1	4.4	19	4.4	7.9e-1141	29.2	6.7	1.2e-486	25.5	5.8	1.7e-412
8. Insulator	CTCF	0.9	2.5	2.7	2e-72	1.9	2.1	2.8e-8	2.7	2.9	3.6e-20
9. Transcription Transition	H3K36me3, H4K20me1, H3K4me1	0.5	1.6	3.0	3.2e-59	1.8	3.3	5.9e-15	1.8	3.4	6.1e-17
10. Transcription Elongation	H3K36me3	4.4	6.2	1.4	8e-28	5.6	1.3	5.4e-4	6.1	1.4	4.5e-7
11. Weak Transcription	none	12.8	12.4	1.0	4.9e-2	10.3	0.8	1.8e-5	9.5	0.7	2.9e-9
12. Repressed	H3K27me3	6.4	11.7	1.8	1.9e-152	10.6	1.7	2.4e-20	10.4	1.6	9.5e-21
13. Heterochrom/lo	none	65.0	42.9	0.7	6.7e-770	33.9	0.5	2.6e-278	33.3	0.5	2.1e-312
14. Repetitive/CNV	medium overall marks	0.1	0.2	1.5	8.4e-3	0.6	4.4	8.3e-8	1.0	6.9	2.1e-18
15. Repetitive/CNV	high overall marks	0.1	0.3	2.5	1.1e-7	0.9	8.8	3.5e-18	1.0	10.4	5.6e-26

1 top CSB peaks include only CSB peaks with rpm above 1, and there are 3302 peaks in total.

2 partial list, for a detailed description please see Ernst et al. (2011) [34].

3 Enrichment: % CSB, top CSB and CSBΔN1 peak coverage divided by % genome-wide occurrence.

To gain insights into the molecular functions of genes that lie close to CSB occupancy sites, we searched for overlaps with the Molecular Signatures Pathways Database (MSigDB) using the Genomic Regions Enrichment of Annotations Tool (GREAT). The top terms associated with occupancy common to CSB and CSBΔN1 involve the roles of epidermal growth factor receptor (EGFR) transactivation by G-protein coupled receptors (GPCR), mechanism of gene regulation by peroxisome proliferators, G-alpha (12/13) signaling, and NFκB activation (Figure 1C).

### ATP hydrolysis by CSB is dispensable for chromatin association during replicative cell growth

**Figure 2:**
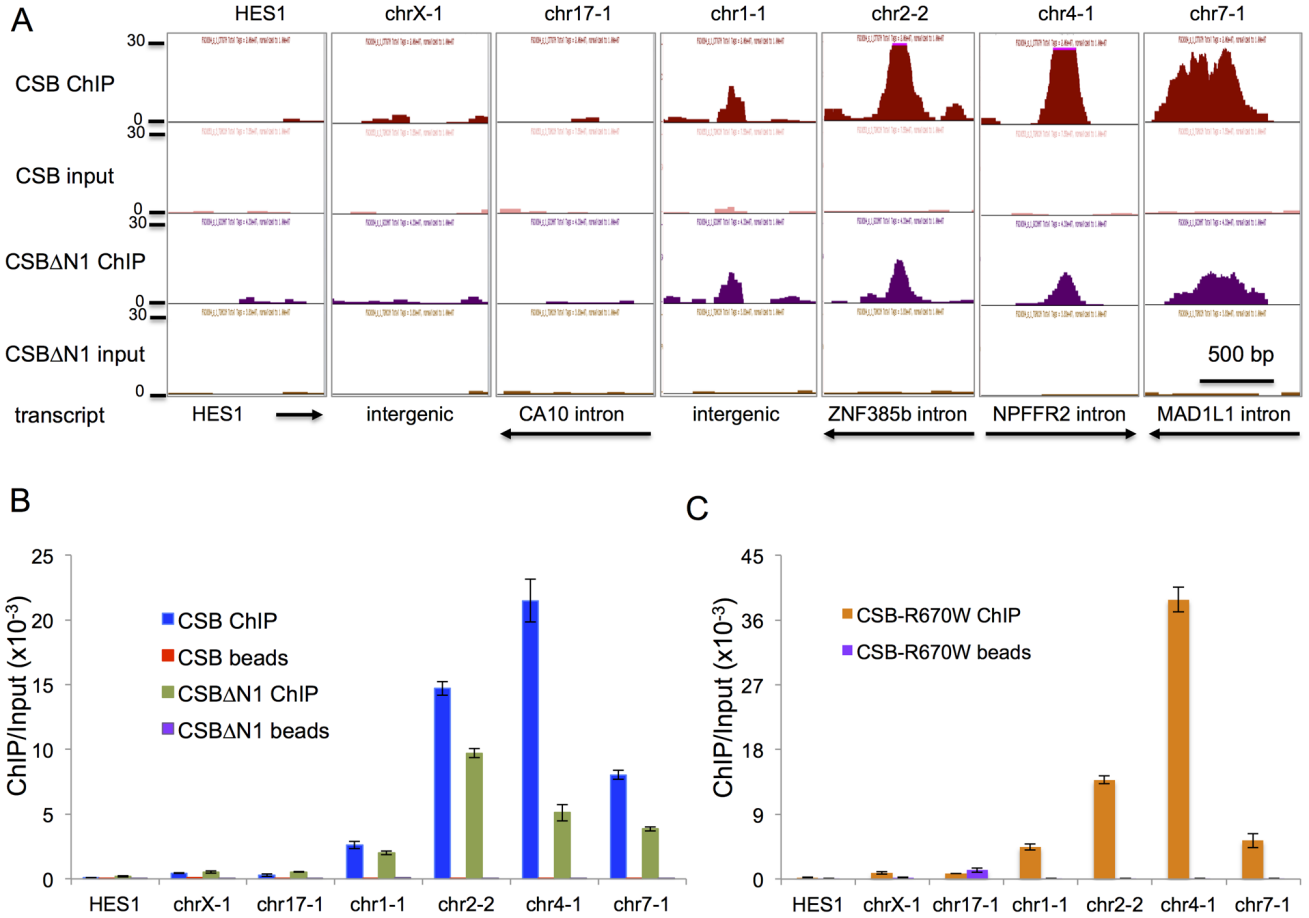
Validation of ChIP-seq results by ChIP-qPCR. (A) Screen shots of ChIP-seq results displayed from the UCSC genome browser. Shown are seven different regions with associated transcripts. The y-axis unit is reads per million (rpm). The x-axis represent the genomic positions in base pairs as follows: HES1_chr3:193,853,385–193,854,474; chrX:73,766,044–73,767,073; chr17:49,769,851–49,771,080; chr1:236,260,208–236,261,267; chr2:180,324,972–180,325,981; chr4:72,977,907–72,978,936; chr7:2,001,209–2,002,238. The first three regions (HES1, chrX-1, and chr17-1) scored as negative for CSB and CSBΔN1 occupancy and the last four regions (chr1-1, chr2-2, chr4-1, and chr7-1) scored as positive for CSB and CSBΔN1 occupancy (ChIPed DNA versus input DNA). The directions of transcription and gene annotation are noted at the bottom. (B) Bar graphs showing CSB and CSBΔN1 ChIP-qPCR results with associated beads-only controls of the seven genomic regions shown in A. (C) Bar graphs showing CSB^R670W^ ChIP-qPCR results with associated beads-only controls of the seven genomic regions shown in A. The primers used in the qPCR assays are listed in Table S5. Shown are means +/− SEM.

To validate our ChIP-seq results, we selected seven regions to analyze by ChIP-qPCR (Figure 2A). chr1-1, chr2-2, chr4-1, and chr7-1 were four regions that were occupied by both CSB and CSBΔN1. As negative control regions, we examined HES1, chrX-1, and chr17-1. ChIP-qPCR confirmed that CSB is highly enriched at chr1-1, chr2-2, chr4-1, and chr7-1 as compared to HES1, chrX-1, and chr17-1 as well as a “beads-only” control (Figure 2B). Moreover, like CSB, CSBΔN1 was also enriched at the same four regions (Figure 2B).

Previously, we found that ATP hydrolysis by CSB is essential for the recruitment of CSB to UV-induced DNA lesions, a necessary and early step in the process of transcription-coupled DNA repair [35]. We, therefore, determined if the ATP hydrolysis activity of CSB is also important for its targeting to specific genomic regions in the absence of UV treatment. To accomplish this, we used a Cockayne syndrome-associated mutant CSB protein, CSB^R670W^, which contains a single amino acid substitution at position 670 [36]. This missense mutation, located within the ATPase domain of CSB, disrupts the ability of CSB to hydrolyze ATP [35]. As shown in Figure 2C, CSB^R670W^, like CSB, was targeted to chr1-1, chr2-2, chr4-1, and chr7-1, but not to the negative control regions (HES1, chrX-1, and chr17-1) (Figure 2C) [35]. These results suggest that ATP hydrolysis by CSB is not critical for the recruitment of CSB to chromatin during replicative cell growth, in contrast to the recruitment of CSB to DNA lesion-stalled transcription upon UV irradiation.

### CSB and CSBΔN1 are enriched at genomic regions containing a TPA-response element

**Figure 3:**
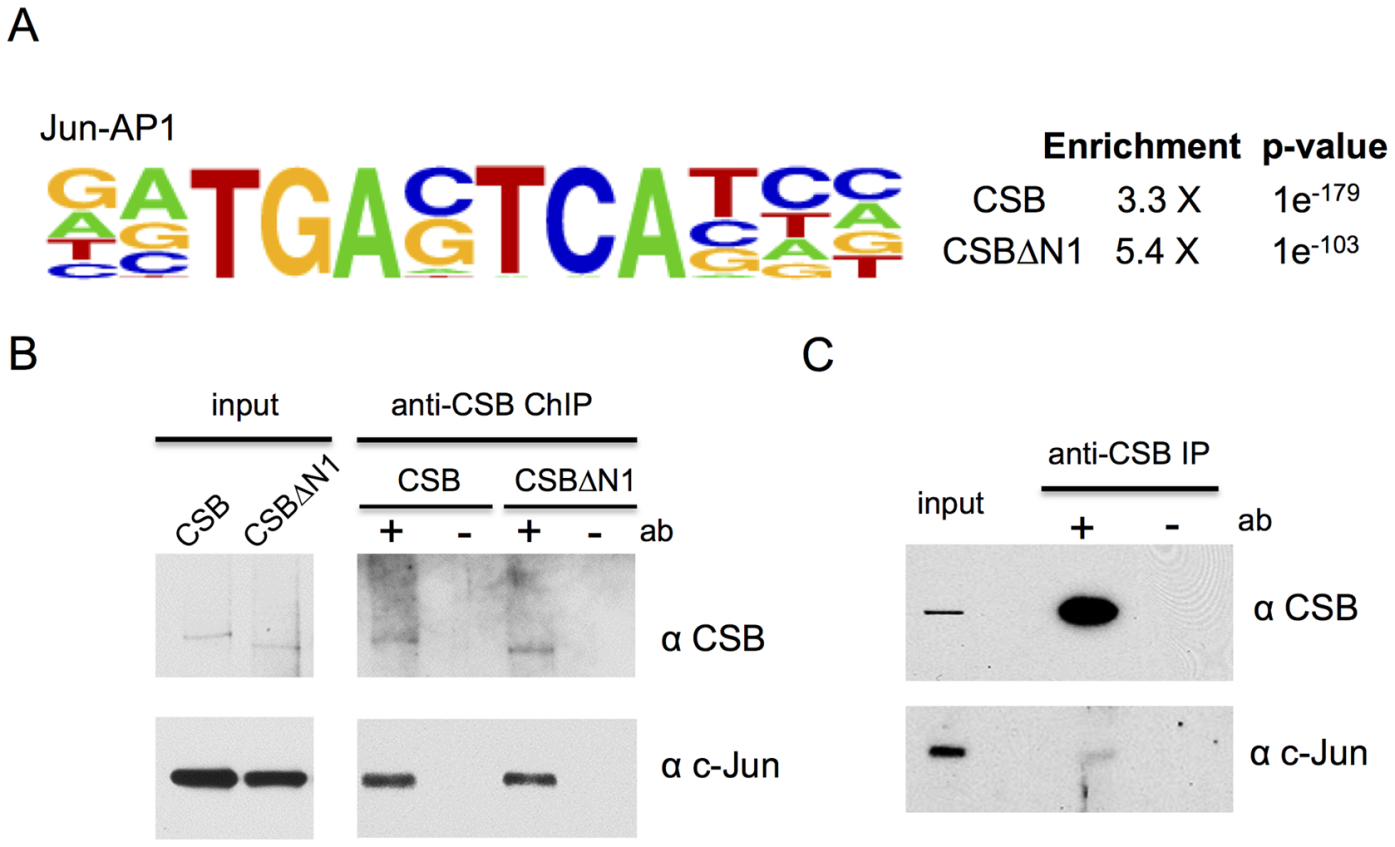
CSB and CSBΔN1 are enriched at sites containing the c-Jun/AP-1 binding motif (TPA-response element). (A) Motif enrichment at CSB and CSBΔN1 occupancy sites. Shown are the fold enrichments with associated p-values. (B) ChIP-western analysis of CSB and CSBΔN1 association with c-Jun on chromatin. (C) Western blot analysis showing c-Jun and CSB co-immunoprecipitation from the soluble (chromatin-free) fraction of cell lysates.

To better understand the mechanisms by which CSB is targeted to specific genomic regions during replicative cell growth, we used HOMER to determine if binding motifs for sequence-specific transcription factors were enriched at sites of CSB occupancy (Figure 3A). This analysis revealed strong enrichment of the TPA (12-O-tetradecanoylphorbol-13-acetate)-response element, TGASTCA (where S denotes a G or C). This motif is most notable for binding AP-1 (activator protein 1) transcription factors that consist of either Jun-Jun homodimers or Jun-Fos heterodimers (Figure 3A) [37], [38]. We classified the CSB occupancy sites that contain TPA-response elements into the same seven functional categories and found that these sites are slightly over-represented at transcription termination sites (TTSs) (1.8% vs. 0.9% for a random genomic sequence, p-value 1.9e-05). CSB occupancy sites containing TPA-response elements were also under-represented at 3′ UTRs (0.7% vs. 1.4%, p-value 9.3e-4) and exons (1.1% vs. 1.9%, p-value 6.1e-04) (Figures S2 and S5).

### The sequence-specific transcription factor c-Jun targets CSB to specific genomic regions

Given that CSB does not bind to DNA in a sequence-specific manner [4], [10], [11], but is enriched at regions containing the TPA-response element, this observation suggested that AP-1 transcription factors might target CSB to specific genomic loci. To test this hypothesis, we first determined if CSB could interact with c-Jun, the most potent transcriptional activator of the Jun protein family [37], [38]. ChIP-western analysis was performed with cells that were pre-extracted to remove soluble CSB and c-Jun protein before formaldehyde cross-linking. As shown in Figure 3B, chromatin immunoprecipitation using an anti-CSB antibody revealed that c-Jun could be found together with either CSB or CSBΔN1 in the same protein-DNA complex. Co-immunoprecipitation analysis of unfixed cell lysates, in which the chromatin fraction had been removed by centrifugation, also revealed an association between soluble CSB and c-Jun, albeit weaker than that observed in the chromatin fraction shown by ChIP-western analysis (compare Figure 3C to 3B).

**Figure 4:**
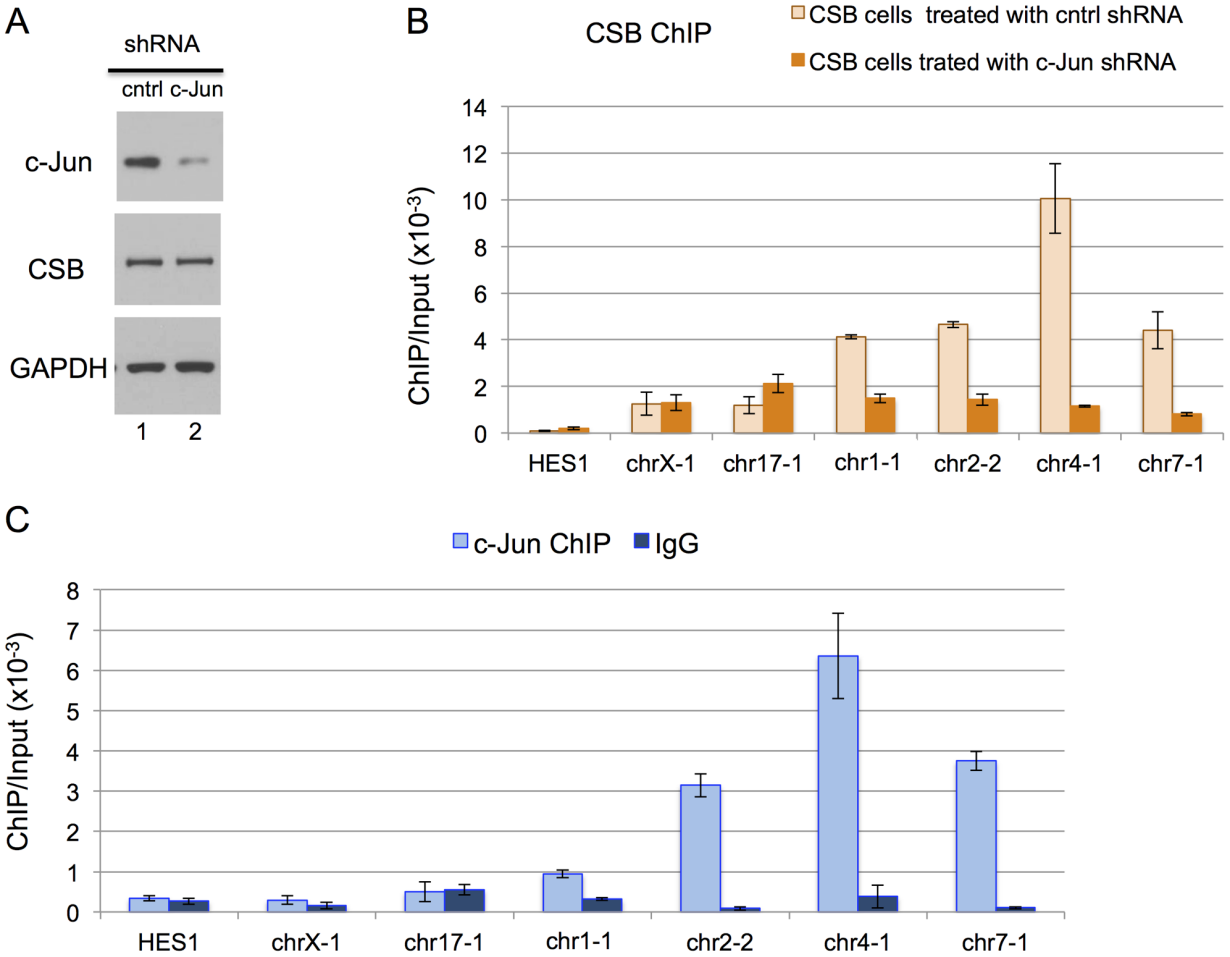
c-Jun is critical for targeting CSB to genomic regions containing the TPA-response element. (A) Western blot showing a significant reduction in c-Jun protein levels in cells expressing c-Jun shRNA. (B) Bar graphs showing CSB ChIP-qPCR results obtained from cells expressing a c-Jun shRNA or a control shRNA. A significant reduction in CSB occupancy occurred at four genomic regions containing the TPA-response element TGASTCA (chr1-1, chr2-2, and chr4-1) or an AP-1 like motif, TGAATCA (chr7-1), only in cells treated with c-Jun shRNA. The seven regions analyzed are as shown in Figure 2. (C) Bar graphs showing c-Jun ChIP-qPCR results, demonstrating c-Jun occupancy at the four genomic regions. Shown are means +/− SEM.

We next used shRNA-mediated RNA interference to directly examine the impact of c-Jun on the targeting of CSB to genomic regions containing a TPA-response element. As shown in Figure 4A, we were able to significantly reduce c-Jun protein levels through c-Jun shRNA expression. We used ChIP-qPCR to compare the recruitment of CSB in cells expressing c-Jun shRNA to cells expressing a control shRNA. As shown in Figure 4B, there was a dramatic decrease in CSB enrichment at regions containing the TGASTCA motif (chr1-1, chr2-2, and chr4-1) or an AP-1-like motif TGAATCA (chr7-1) in cells expressing c-Jun shRNA as compared to the control shRNA. On the other hand, there were no significant changes in the enrichment of CSB at the negative control regions (HES1, chrX-1, and chr17-1). To demonstrate that c-Jun does, indeed, occupy these four genomic regions (chr1-1, chr2-2, chr4-1, and chr7-1), we performed anti-c-Jun ChIP followed by qPCR. As shown in Figure 4C, c-Jun was enriched at each of these loci. Taken together, these results indicate that CSB can be targeted to genomic sites containing a TPA-response element through an association with a c-Jun-containing AP-1 transcription factor.

CSB-PGBD3 is a fusion protein that arises from alternative splicing between sequence encoding the N-terminal 465 amino acids of CSB with sequence encoding a piggybac transposase that lies within the fifth intron of the CSB gene [30], [39]. The genomic sites of CSB-PGBD3 occupancy had been previously determined in UVSS1KO cells (a CSB and CSB-PGBD3 null cell line) that had been reconstituted with CSB-PGBD3 [39]. Given that both CSB and the CSB-PGBD3 fusion protein interact with c-Jun and are targeted to TPA-response elements, we determined the overlap in CSB and CSB-PGBD3 occupancy. To accomplish this, we used HOMER to analyze the published CSB-PGBD3 ChIP-seq data with the same parameters used to analyze the CSB ChIP-seq data, and we identified 1,590 CSB-PGBD3 ChIP-seq peaks. Of those peaks, 165 were common to CSB and CSB-PGBD3 (Tables 3 and S3). And, of the common peaks, 45% contained a TPA-response element.

**Table 3:** Comparison of CSB and CSBΔN1 occupancy with CSB-PGBD3#.

ChIP-seq	No. of common occupancy sites with CSB-PGBD3#	Percent common occupancy of CSB or CSBΔN1 with CSB-PGBD3	Percent common occupancy of CSB-PGBD3 with CSB or CSBΔN1$	No. of c-Jun/AP-1 motifs in common occupancy	p-values§ ^,^ † ^,^ ‡
CSB	165	0.9%	10.4%	74 (45%)	3.5e-10§ 4.6e-38†
CSBΔN1	81	2.2%	5.1%	42 (52%)	2.1e-8§ 6.7e-19‡

# CSB-PGBD3 ChIP-seq data are from Gray et al. (2012) [39].

$ Total number of CSB-PGBD3 occupancy sites is 1590.

§ hypergeometric p-value against CSB-PGBGD3 peaks.

† hypergeometric p-value against CSB peaks.

‡ hypergeometric p-value against CSBΔN1 peaks.

Gene ontology analysis using GREAT revealed the top three “Biological Processes” associated with common CSB and CSB-PGBD3 occupancy sites were tissue development, positive regulation of developmental processes and positive regulation of catecholamine secretion (Table 4). GREAT was also used to compare human genes enriched for CSB and CSB-PGBD3 to genes involved in mouse phenotypes. Of interest, the top three mouse phenotypes associated with common CSB and CSB-PGBD3 occupancy were decreased body weight, abnormal body weight and decreased bone marrow cell number (Table 4). Whether or not deregulation of the genes occupied by both CSB and CSB-PGBD3 contribute to the clinical features associated with Cockayne syndrome awaits further studies.

**Table 4:** GO analysis of common CSB and CSB-PGBD3 occupancy.^1^

Term Name	Binomial Raw P-Value
Go Biological Process	
Tissue development	8.40E-07
Positive regulation of developmental process	1.80E-06
Positive regulation of Catecholamine secretion	4.30E-06
Mouse Phenotype	
Decreased body weight	3.40E-08
Abnormal body weight	4.50E-08
Decreased bone marrow cell number	3.30E-07

1 Analysis was performed using GREAT (v.2.0.2).

### CSB can regulate nearby transcription

We next determined the extent to which CSB occupancy can impact the expression of nearby genes. Using reverse transcription coupled with quantitative PCR (RT-qPCR), we compared RNA expression levels of 10 genes, which displayed significant CSB occupancy, in CS1AN-sv cells expressing CSB to those in CS1AN-sv cells harboring an empty vector [8]. For these experiments, RNA expression was normalized to β-actin transcript levels.

**Figure 5:**
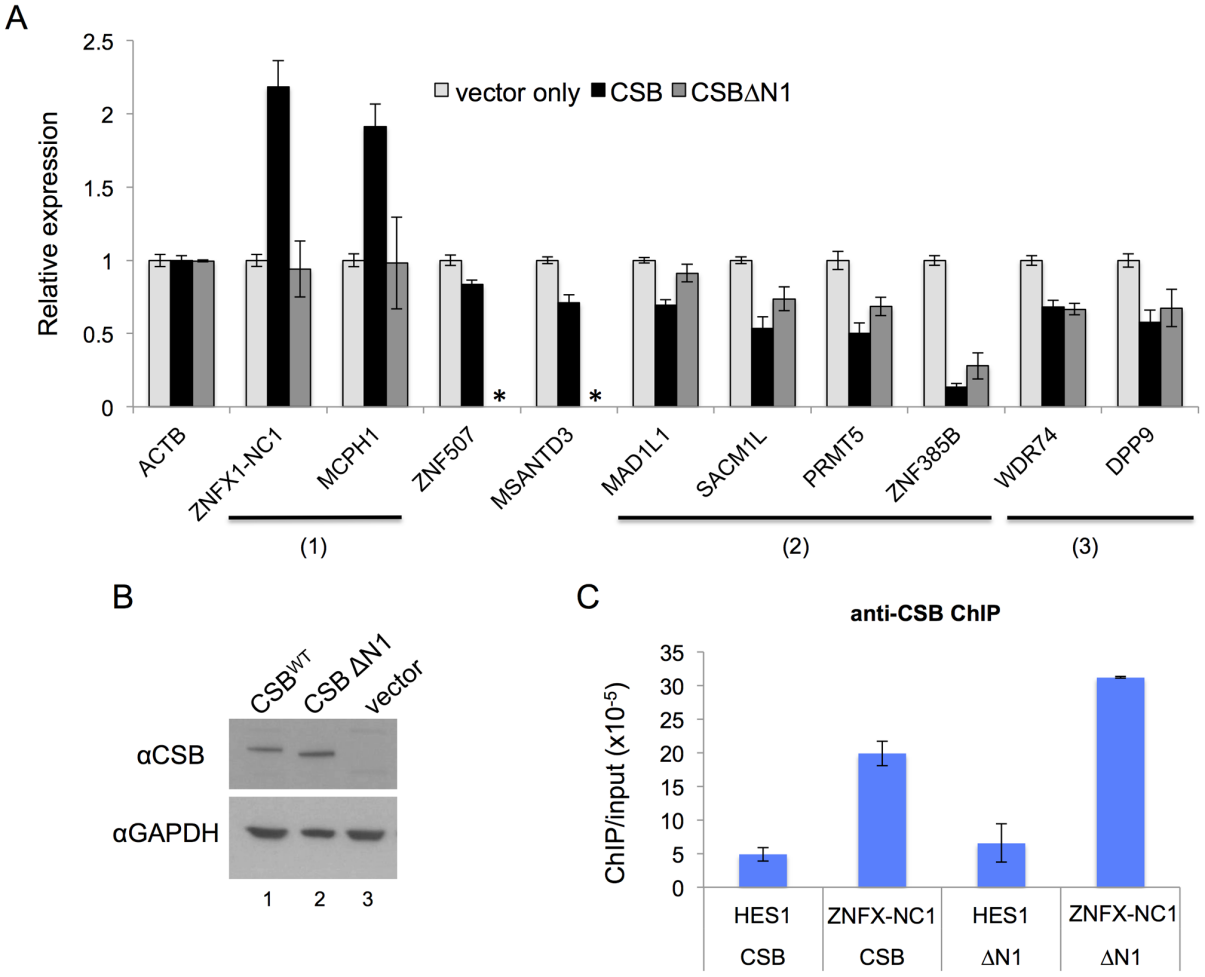
CSB can influence nearby gene expression and has both remodeling-dependent as well as independent functions. (A) Gene expression was assayed by RT-qPCR using CS1AN-sv cells expressing CSB, CSBΔN1, or harboring an empty vector, to determine the effect of CSB and CSBΔN1 on the expression of CSB-occupied genes. Expression levels were normalized to β-actin (ACTB) and are presented as fold change over the vector only control. Shown are means +/− SEM. All data are averaged from six biological replicates, and each biological replicate consisted of three technical replicates. The expression of ten genes was analyzed (see text for the relative positions of CSB occupancy at these genes). The primers used in RT-qPCR assays are listed in Table S6. CSBΔN1 could not enhance expression of the ZNFX1-NC1 or MCPH1 genes, unlike CSB (1), while CSBΔN1 could partially substitute for CSB function at the MAD1L1, SACM1L, PRMT5 and ZNF385B genes (2). CSBΔN1 could fully substitute for CSB function at the WDR74 and DDP9 genes (3). CSBΔN1 was not examined for ZNF507 or MSANTD3 (*). (B) Western blot analysis demonstrating that the levels of CSB and CSBΔN1 expression in the stable cell lines that were used for these assays were similar. (C) ChIP-qPCR reveals that both CSB and CSBΔN1 were recruited to ZNFX1-NC1 (chr20-1) locus. HES1 was used as a negative control region in this assay.

ZNFX-NC1 and MCPH1 are two examples of genes that were positively regulated by CSB (Figure 5A). The ZNFX-NC1 gene expresses a noncoding RNA that is subsequently processed into three snoRNAs and is involved in cell proliferation and differentiation [40], and our ChIP-seq results revealed that CSB binds to the first exon/intron junction of this gene. CSB is also associated with an intronic region of the microcephalin 1 (MCPH1) gene, which encodes a DNA damage response protein that may be involved in neurogenesis and the regulation of cerebral cortex size [41]. RT-qPCR revealed that re-introducing CSB into CS1AN-sv cells increased the expression of ZNFX-NC1 and MCPH1 about two-fold relative to the vector-only control.

Also shown in Figure 5A are examples of eight genes that are negatively regulated by CSB. ZNF507 and ZNF385B are two zinc finger proteins likely involved in transcription regulation [42], [43]. MSANTD3 is a Myb/SANT-like DNA-binding domain containing protein and is associated with brain tumors [44]. MAD1L1 (Mitotic Arrest Deficient-Like 1) is a component of the spindle assembly checkpoint [45]. SACM1L (suppressor of actin mutations 1-like) is a phosphoinositide phosphatase, which degrades phosphoinositides and plays a key role in signal transduction events [46]. PRMT5 is a protein arginine methyltransferase and plays a role in early development and pluripotency [47]. WDR74 (WD Repeat Domain 74) likely plays an essential role in RNA transcription, stability, and/or processing [48]. DPP9 (dipeptidyl peptidase 9) regulates signaling pathways that affect cell survival and proliferation [49]. Among these genes, CSB occupies the intronic regions of ZNF507, MAD1L1 and ZNF385B, and the promoter regions of MSANTD3, SACM1L, PRMT5, WDR74, and DPP9. RT-qPCR analysis revealed that CSB decreased expression of these genes between 10% and 85%. Altogether, our results revealed that CSB can both positively and negatively regulate the expression of genes that lie adjacent to its occupancy sites.

To identify, on a global level, potential direct transcriptional targets of CSB, we compared our ChIP-seq data to publicly available CSB microarray data [28], [29]. CSB-dependent transcription profiling had been performed in both hTERT immortalized CS1AN fibroblasts (as compared to SV40-transformed CS1AN cells used in our ChIP-seq study) and SV40 transformed UVSS1KO fibroblasts.

In the CS1AN/hTERT cells, Newman et al. (2006) identified 188 CSB up-regulated genes and 205 down-regulated genes. As shown in Table 5 and S4, 37% of the up-regulated genes and 14% of the down-regulated genes were occupied by CSB; among these genes, 9% and 4%, respectively, were associated with TPA-response elements. In the UVSS1KO study, Bailey et al. (2012) identified 100 CSB up-regulated genes and 184 down-regulated genes [29]. As shown in Table 6, 33% of the up-regulated genes and 34% of the down-regulated genes were occupied by CSB (Tables 6 and S4); among them, 10% and 7%, respectively, were associated with TPA-response elements. Taken together, these analyses suggest that at least 25% of the CSB-mediated gene expression changes might directly result from CSB occupancy.

**Table 5:** Correlation of CSB ChIP-seq data with CSB transcription profiling data of Newman et al. (2006).

	Number of genes^1^	Number of genes associated with CSB occupancy	Number of CSB- binding sites associated with CSB-responsive genes^2^	Number of c-Jun/AP-1 motifs^3^
Up-regulated by CSB	188	69 (37%)	196 (1.1%) (p-value: <1.e-300)	18 (9%) (p-value: 0.08)
Down-regulated by CSB	205	28 (14%)	45 (0.25%) (p-value: 1)	2 (4%) (p-value: 0.17)

1 CSB transcription profiling data are from Newman et al. (2006) [28].

2 p-values were calculated using a z-score after randomly generating 17,779 peaks against the genome.

3 Hypergeometric p-values were calculated against the total number of CSB ChIP-seq peaks (this study).

**Table 6:** Correlation of CSB ChIP-seq data with CSB and CSB-PGBD3 transcription profiling data of Bailey et al. (2012).

	Number of genes^1^	Number of genes associated with CSB occupancy	Number of CSB- binding sites associated with CSB-responsive genes^2^	Number of c-Jun/AP-1 motifs^3^
Up-regulated by CSB	100	33 (33%)	73 (0.4%) p-value: 0.67	7 (10%) p-value: 0.13
Down-regulated by CSB	184	63 (34%)	182 (1%) p-value: 0.99	12 (7%) p-value: 0.09
Up-regulated by CSB-PGBD3	248	48 (19%)	81 (0.5%) p-value: 0.27	12 (15%) p-value:0.014
Down-regulated by CSB-PGBD3	273	81 (30%)	307 (1.7%) p-value: 0.99	26 (8%) p-value: 0.07
Up-regulated by CSB+CSB-PGBD3	913	201 (22%)	608 (3.4%) p-value: 1	62 (10%) p-value: 0.006
Down-regulated by CSB+CSB-PGBD3	329	98 (30%)	297 (1.7%) p-value: 1	19 (6%) p-value: 0.17

1 Transcription profiling data are from Bailey et al. (2012) [29].

2 p-values were calculated using a z-score after randomly generating 17,779 peaks against the genome.

3 Hypergeometric p-values were calculated against the total number of CSB ChIP-seq peaks (this study).

The Bailey et al. (2012) study also examined the transcription profile of USS1KO cells coexpressing CSB and CSB-PGBD3 as well as USS1KO cells expressing CSB-PGBD3 alone. When we compared the CSB occupancy data with those transcription profiling data (Tables 6 and S4), we found that 22% of the genes up-regulated and 30% of genes down-regulated by co-expression of CSB and CSB-PDGB3 were associated with CSB occupancy, and 19% of the genes up-regulated by CSB-PGBD3 alone and 30% of the genes down-regulated by CSB-PGBD3 alone were associated with CSB occupancy. These observations are consistent with the hypothesis that CSB and CSB-PGBD3 may work together to regulate the transcription of certain genes [29]. Additionally, TPA-response elements were significantly enriched at CSB-occupied genes that were transcriptionally upregulated by both CSB and CSB-PGBD3 or by CSB-PGBD3 alone.

### CSB has chromatin remodeling-dependent and -independent activities in transcription regulation

To determine whether the remodeling activity of CSB is required for its function in transcription regulation, we used RT-qPCR to examine the effect of the remodeling defective CSBΔN1 protein on RNA expression [8]. For this analysis, we focused on genes that were occupied by both CSB and CSBΔN1, as revealed by the ChIP-seq analysis. Western blot analysis using an antibody that recognizes the C-terminal region of CSB demonstrated that the levels of CSB or CSBΔN1 expression in the stable CS1AN-sv cell lines used for this analysis were similar (Figure 5B). Additionally, immunofluorescence examination of those cell lines revealed that the number of cells expressing CSB or CSBΔN1 were similar (>95%, data not shown). From this analysis, we found that expression of the ZNFX-NC1 gene, which was enhanced by CSB, was not enhanced by CSBΔN1 (Figure 5A); the presence of CSB resulted in a greater than two-fold increase in ZNFX-NC1 RNA levels, while cells expressing CSBΔN1 had ZNFX-NC1 RNA levels similar to that of CS1AN-sv cells harboring an empty vector. ChIP-qPCR confirmed that both CSB and CSBΔN1 were recruited to the ZNFX-NC1 locus (Figure 5C). The other genes that we examined showed decreased RNA expression levels in response to CSB expression (Figure 5A). Some of the transcript levels were also decreased by CSBΔN1, but to different degrees (Figure 5A). These results suggest that the chromatin remodeling activity of CSB is important for transcription regulation of some genes (Figure 5A, groups (1) and (2)) and dispensable for the regulation of others (Figure 5A, group (3)). These results also reveal a chromatin remodeling-independent activity of CSB in transcription regulation. Of note, CSBΔN1, unlike CSB, did not impact MCPH1 gene expression (Figure 5A). Given that we did not see significant enrichment of CSBΔN1 at this locus (data not shown), we cannot distinguish if the chromatin remodeling activity or another CSB-related function is important for up-regulating MCPH1 gene expression (Figure 5A).

**Figure 6:**
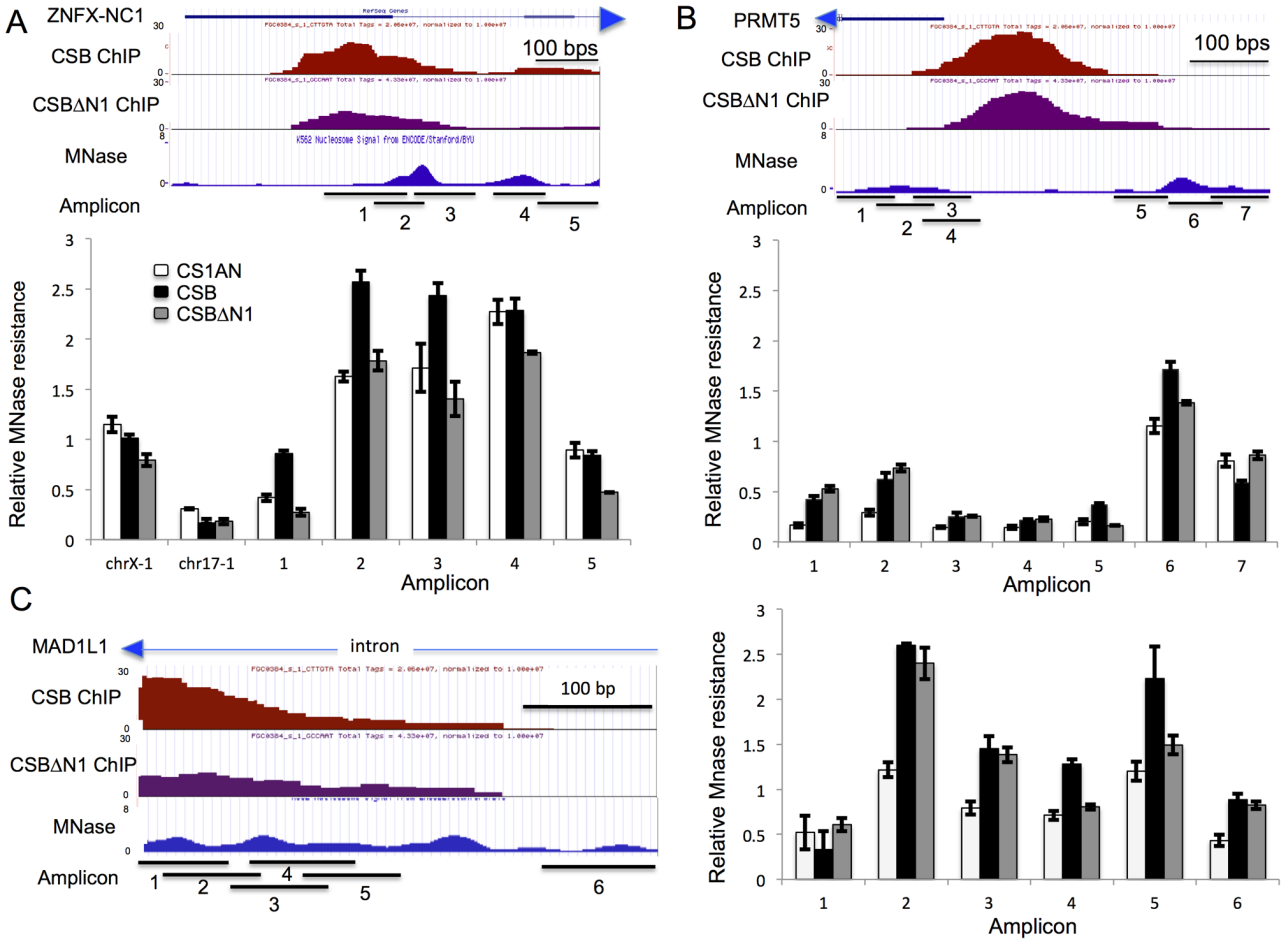
CSB can alter the MNase sensitivity of nearby nucleosomes. (A–C) first panels from top to bottom: screen shots from the UCSC genome browser (GRCh37/hg19 Assembly) showing the RefSeq gene and direction of transcription (arrow head), the position of CSB occupancy (CSB ChIP), the position of CSBΔN1 occupancy (CSBΔN1 ChIP), ENCODE MNase-seq data obtained from K562 cells (MNase), and the amplicons used in the MNase-qPCR assays (Amplicon). The chromosome coordinates shown are (A) Chr20:47,894,930–47,895,599 (B) Chr14:23,398,657–23,399,208 and (C) Chr7:2,001,747–2,002,144. Second panels in A–C are bar graphs showing results from MNase-qPCR assays. The primers used in the MNase-qPCR assays are listed in Table S7. Shown are means +/− SEM.

To further demonstrate that the chromatin remodeling activity of CSB is important for regulating ZNFX-NC1 expression, we performed micrococcal nuclease (MNase) sensitivity assays. MNase is a nuclease that preferentially digests naked DNA and leaves nucleosomal DNA intact. ENCODE data obtained from the extensively studied K562 and GM12878 cell lines indicates that there are two MNase-resistant regions adjacent to the CSB occupancy site in the ZNFX-NC1 gene (Figure 6A) [33]: one MNase-resistant region is centered at position 47,895,320 on chromosome 20 and a less resistant region about 150 bp downstream (Figure 6A). To determine the impact of CSB on local chromatin structure, we treated formaldehyde cross-linked cells with limiting amounts of MNase and isolated mononucleosomal DNA (∼150 bp) (Figure S6). Quantitative PCR using primer sets that span the ∼670 bp region surrounding the CSB occupancy site were used to compare differences in MNase sensitivity among cells not expressing CSB (CS1AN), expressing wild-type CSB, or expressing the remodeling-defective CSBΔN1 protein. All PCR reactions were normalized to naked genomic DNA. ChrX-1 and chr17-1 were used as two negative control regions, as they are not occupied by CSB.

As shown in Figure 6A, cells expressing CSB or CSBΔN1, as well as cells harboring an empty vector, had similar levels of amplicon enrichment from chr17-1 and chrX-1, indicating similar MNase sensitivities. However, cells expressing CSB demonstrated a greater enrichment of amplicons 1, 2, and 3 than cells expressing CSBΔN1 or harboring an empty vector (CS1AN). These results indicate that this region is more protected from MNase digestion in CSB expressing cells. On the other hand, enrichment of amplicons 4 and 5 was very similar between CSB and CS1AN, while CSBΔN1 displayed slightly less MNase resistance. Given that chromatin remodeling by CSB is required for transcription up-regulation of ZNFX-NC1 (Figure 5A), our MNase-qPCR analyses suggest that CSB binds to the promoter of ZNFX-NC1 and enables the region that spans amplicons 1–3 to become more MNase-resistant through nucleosome repositioning or nucleosome assembly, which promotes transcription up-regulation of ZNFX-NC1.

We next examined the MNase sensitivity of a region of the PRMT5 promoter near a CSB occupancy site. Remodeling by CSB is partially required for suppression of PRMT5 expression (Figure 5A). As depicted in Figure 6B, there are two MNase-resistant regions that lie on either site of the CSB occupancy site. The nucleosome to the right appears to be better positioned than the nucleosome to the left. As shown in Figure 6B, cells expressing either CSB or CSBΔN1 had similar sensitivity to MNase at the region covered by amplicons 1 and 2, but both displayed greater resistance to MNase than CS1AN cells. The region covered by amplicons 3–5 appeared to be more sensitive to MNase, suggesting a relatively more open chromatin structure. Examining MNase sensitivity patterns at the regions covered by amplicons 6 and 7, we found that cells expressing CSB showed greater MNase resistance to the region covered by amplicon 6 than cells not expressing CSB (CS1AN). Interestingly, cells expressing CSBΔN1 demonstrated an intermediate enrichment of amplicon 6 (less than cells expressing CSB but more than CS1AN cells). On the other hand, enrichment of amplicon 7 was very similar between cells expressing CSBΔN1 and CS1AN cells, while cells expressing CSB displayed less MNase resistance. Together, these observations suggest that CSB-expressing cells have a better-positioned nucleosome around amplicon 6. Given that CSB suppressed PRMT5 more efficiently than CSBΔN1 (Figure 5A), these results suggest that a better-positioned nucleosome at the region of the PRMT5 promoter covered by amplicon 6 may facilitate PRMT5 repression.

We also examined MNase sensitivity near the site of CSB occupancy in an intron of the MAD1L1 gene, where several MNase-resistant regions were predicted based on ENCODE data [33]. RT-qPCR analysis revealed that the remodeling activity of CSB was partially required for suppression of MAD1L1 expression (Figure 5A). As shown in Figure 6C, cells expressing CSB demonstrated a similar MNase resistance to cells expressing CSBΔN1 at the region covered by amplicons 2, 3, and 6, but higher than CS1AN cells. Additionally, CSB-expressing cells displayed greater MNase sensitivity at the region covered by amplicons 4 and 5 than CSBΔN1 or CS1AN cells, which were similar to each other. These results suggest that increased nucleosome occupancy at the region of the MAD1L1 gene covered by amplicons 4–5 may facilitate MAD1L1 repression.

Lastly, we examined MNase sensitivity at the promoter of the WDR74 gene, where remodeling by CSB was dispensable for CSB-dependent suppression of WDR74 expression. Cells expressing CSB or CSBΔN1 displayed very similar patterns of MNase sensitivity, agreeing with the results of our RT-qPCR analysis (Figure 5A), which revealed that CSB and CSBΔN1 had a similar effect on WDR74 expression (Figure S7). Interestingly, the nucleosome structure at the promoter of the WDR74 gene appeared to be relatively more open, as the overall MNase-qPCR signals were lower than those obtained from the ZNFX-NC1, PRMT5 and MAD1L1 genes (Figure 6). A more open nucleosome structure could account for, at least in part, our observation that suppression of WDR74 expression by CSB does not rely upon remodeling activity of CSB.

## Discussion

Our results reveal that the mechanism that targets CSB to chromatin for transcription regulation is distinct from the mechanism that targets CSB during transcription-coupled DNA repair [35]. The association of CSB with specific genomic loci during replicative cell growth does not rely upon ATP hydrolysis by CSB (Figure 2C); however, ATP hydrolysis by CSB is essential for its targeting to sites of UV-induced DNA damage [35]. Therefore, stable chromatin association during transcription-coupled DNA repair appears to be an active process while stable chromatin association during transcription regulation appears to be a passive process. Current evidence suggests that during transcription-coupled DNA repair, ATP hydrolysis by CSB induces a conformational change that exposes a chromatin interaction surface, which is normally occluded by the N-terminal region of CSB [35]. Given that the association of CSB with chromatin in the absence of UV-induced DNA lesions does not rely upon ATP hydrolysis, this would suggest that the residues that mediate the c-Jun association are normally exposed.

**Figure 7:**
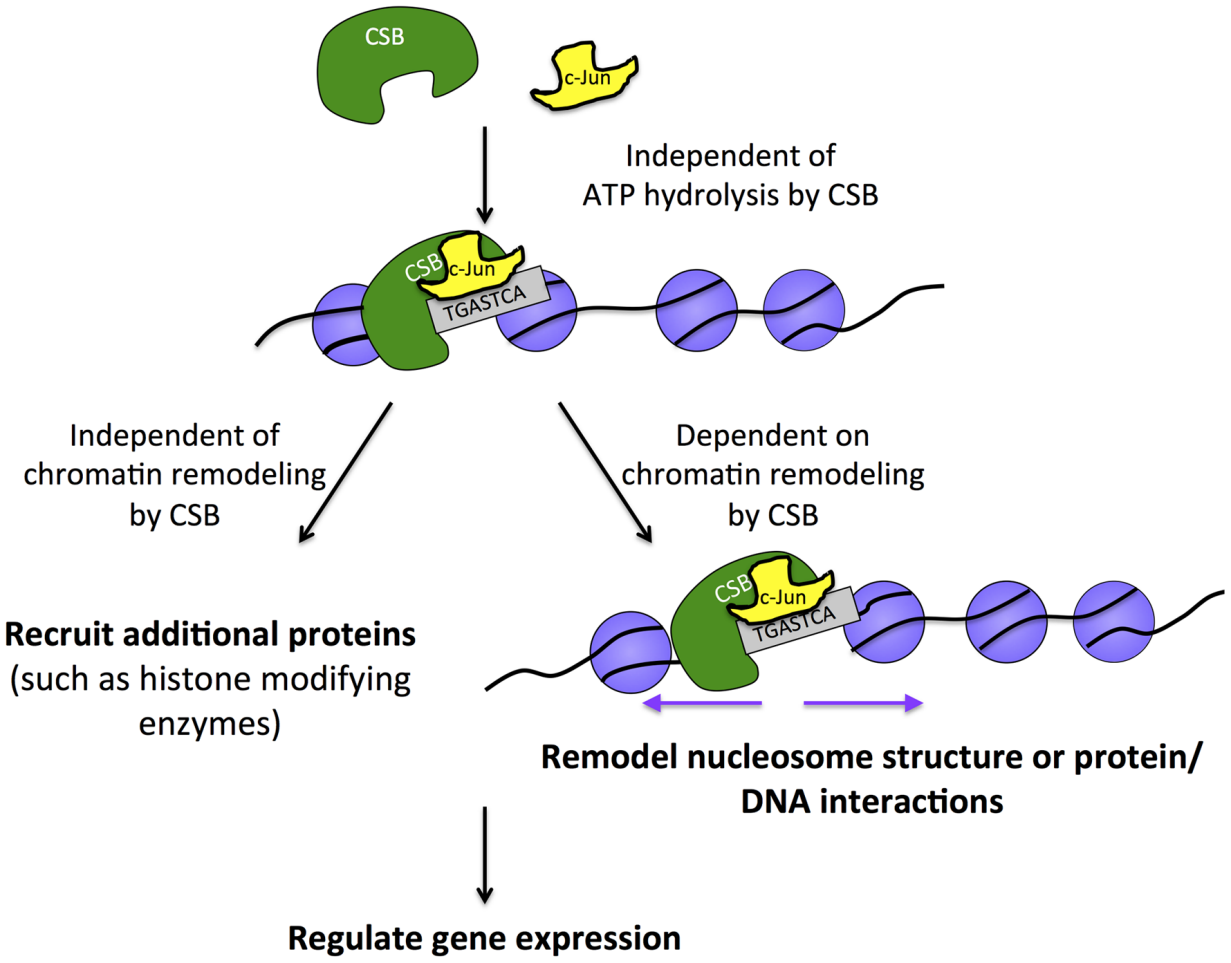
Model for the targeting of CSB to chromatin for transcription regulation. The sequence-specific transcription factor c-Jun interacts with CSB (directly or indirectly) and either delivers CSB to chromatin or is pre-bound and stabilizes the association of CSB with chromatin at regions containing the TPA-response element. This targeting mechanism is independent of ATP hydrolysis by CSB and, therefore, distinct from CSB targeting to chromatin in response to UV irradiation, which requires ATP hydrolysis. Once chromatin associated, CSB can either activate or repress the expression of nearby genes. The function of CSB in regulating gene expression relies upon both remodeling-dependent and remodeling-independent activities of CSB.

Recently, Gray et al. showed that the N-terminal region of CSB mediates the interaction between c-Jun and CSB-PGBD3 [39]. Based upon this observation and the knowledge that the CSB-PGBD3 fusion protein contains the N-terminal 465 amino acids of CSB, it is likely that the N-terminal 465 amino acids also mediates the association of full-length CSB with c-Jun. However, an interaction between full-length CSB and c-Jun was not detected in that study, although a robust interaction between CSB-PGBD3 and c-Jun was observed [39]. In agreement with that observation, we observed only modest association between endogenous CSB and c-Jun in chromatin-free cell lysates (Figure 3C). However, a greater degree of association was observed when we specifically examined chromatin-bound proteins (Figure 3B). These observations suggest that the CSB-c-Jun association may be preferentially established or stabilized on chromatin (Figure 7).

CSB and CSB-PGBD3 both interact with c-Jun, and 45% of peaks common to CSB and CSB-PGBD3 contain a TPA-responsive element (Table 3) [39]. These observations are consistent with results obtained from transcription profiling studies, in which it was observed that CSB and CSB-PGBD3 can co-regulate the expression of certain genes [29]. These results further suggest that AP-1 transcription factors might play a crucial role in modulating this co-regulation. Additionally, these results are also consistent with the notion that CSB regulates many genes independently of CSB-PGBD3 [29].

From comparisons between ChIP-seq and transcription profiling data, it can be seen that the majority of CSB occupancy sites are not associated with known CSB-responsive genes (Tables 5–6). This could arise from the different cell lines used in these studies and/or the different immortalization methods used to obtain these cell lines. It is also possible that some CSB-responsive genes were not covered by the microarrays used for the transcription profiling studies and, therefore, complementary approaches such as RNA-seq might offer additional insights, such as the influence of CSB on non-coding RNA expression. Furthermore, our analysis indicates a strong correlation between sites of CSB occupancy and chromatin regions that contain epigenetic signatures of enhancers (Table 2), and many of the CSB peaks (41%, p-value of 6.7e-1536) have DNase I hypersensitive sites lying within 100 bp, as judged by the digital DNase I hypersensitivity clusters in 125 cell lines [33]. These observations suggest that some of the intergenic CSB occupancy sites could function as enhancer elements that might lie at a great distance from their target genes.

The composition of the dimeric AP-1 transcription factors that bind to TPA-response elements varies; for instance, there are three Jun and four Fos family members, and some of these members have variants that result from alternative splicing [37], [38]. Future experiments will unveil the full extent to which different AP-1 complexes can target CSB and how this targeting might be modulated in different cellular contexts. AP-1 participates in a number of fundamental cellular processes, including cell proliferation and cell death. It is tempting to speculate that loss of CSB activity might, to some extent, compromise AP-1-mediated gene regulation, which in turn might contribute to the underlying mechanisms of Cockayne syndrome.

Approximately 15% of the total CSB occupancy sites contain TPA-response elements. The mechanism that underlies the targeting of CSB to regions of the genome that do not contain TPA-response elements is not yet clear, but it is likely that CSB is delivered to or stabilized at these regions through associations with other DNA-binding proteins. A recent study examining the targeting of Isw2 in *Saccharomyces cerevisiae* revealed that this remodeler is primarily targeted to specific loci by sequence-specific transcription factors; however, more than half of the transcription factor-dependent occupancy sites did not contain a cognate binding motif [50]. Chromatin conformation capture suggested that DNA looping between regions that contain a transcription factor binding site with regions that do not is an integral component of the Iswi2 targeting mechanism [50]. Accordingly, DNA looping may also play an important role in the targeting of the CSB remodeler to sites that do not contain a TPA-response element. Interestingly, AP-1, in conjunction with NFκB, was found to mediate DNA looping to regulate gene expression in macrophages [51]. Future studies examining CSB-containing protein complexes and higher-order chromatin structure will offer insights into other CSB targeting mechanisms.

Our ChIP-seq data revealed that 36% of the CSB peaks were unique to CSB and 24% of the CSBΔN1 peaks were unique to CSBΔN1 (Figure 1B and Table 1). We do not yet know the reason underlying this difference. It is possible that some of the occupancy sites unique to CSBΔN1 might represent the initial sites of CSB binding to chromatin and that CSB would subsequently translocate away from these sites during chromatin remodeling, which in turn might contribute to some of the unique CSB peaks. Alternatively, but not mutually exclusive, some of the unique CSB peaks might represent targeting that relies upon functions related to the N1 region, which is deleted in the CSBΔN1 protein, such as mediating protein-protein interactions. Of interest, CSBΔN1 is more significantly enriched at promoters and 5′ UTR regions than CSB (Figures S2, S3).

By examining the effect of CSB on several genes that lie close to CSB occupancy sites, we provide evidence that CSB can directly influence local gene expression mediated by RNA polymerase II. CSB is a member of the SWI2/SNF2 ATP-dependent chromatin remodeling protein family and displays ATP-dependent chromatin remodeling activity *in vitro*; therefore, CSB likely repositions nucleosomes [8], [13] and/or other protein factors [17] to regulate transcription. By mapping nucleosome positions at the ZNFX-NC1, PRMT5 and MAD1L1 genes with MNase, we found that regions adjacent to the CSB occupancy sites are more resistant to MNase digestion in cells expressing CSB than in cells that do not express CSB or express the remodeling-defective CSBΔN1 protein. These results indicate that CSB alters chromatin structure in an ATP-dependent manner to regulate transcription (Figures 6–7). Furthermore, CSB appears to make the border of nucleosome-free regions more pronounced (Figure 6A amplicon 2–3 and Figure 6B amplicon 6), resembling the function of yeast Iswi2 in regulating the length of nucleosome-free regions to prevent cryptic transcription and regulate gene expression [52]. By creating a better-positioned nucleosome (Figure 6C, amplicon 4–5), CSB could also support either transcription activation or repression by preventing the binding of transcriptional repressors or activators, respectively, to the DNA occupied by the nucleosome [53]. Interestingly, in collaboration with NAP1-like histone chaperones, CSB has been shown to efficiently move histone octamers to the center of a DNA fragment *in vitro* [8]. It will be of great interest to investigate the function of NAP1-like chaperones in CSB-mediated transcription regulation. Additionally, the complete rescue of gene expression by the CSBΔN1 protein at certain genes (e.g. WDR74) suggests additional CSB functions in transcription regulation; such functions could include protein recruitment through remodeling-independent mechanisms [12], [54] or protein eviction [55], which may not rely upon the N1 region.

During replicative cell growth, approximately 10% of CSB associates with chromatin, and this likely represents the CSB population that participates in normal transcription regulation. However, in the presence of UV-induced DNA damage (>25 J/m^2^), approximately 90% of the CSB population can become stably associated with chromatin [35]. A fraction of these chromatin-associated CSB molecules would be stabilized at sites of DNA lesion-stalled transcription to participate in DNA repair. In addition, some of these CSB molecules would also be expected to localize to new transcriptional targets, as CSB has been implicated in UV-induced transcription regulation [17]. Additional ChIP-seq analysis of CSB in cells challenged with UV irradiation will reveal if the fraction of CSB that is used during normal transcription regulation is redistributed in response to UV irradiation, either for DNA repair or UV-induced transcription regulation.

Taken together, the results of this study reveal that the CSB remodeler binds to specific regions of the genome to regulate chromatin structure and RNA polymerase II-mediated gene expression. These observations are consistent with the hypothesis that Cockayne syndrome might be, at least in part, a disease of transcription deregulation [28], [29], [56]. Moreover, the results of this study open up new avenues to explore the mechanisms that might contribute to the diverse features of Cockayne syndrome.

## Materials and Methods

### Cell culture

CS1AN-sv cells were maintained in DMEM-F12 with 10% FBS. The CS1AN primary cells have mutations in both CSB alleles, but only one of these alleles was retained after SV40 immortalization [19]; the resulting CS1AN cell line is, therefore, hemizygous for CSB. The retained allele contains an A to T transversion at position 1088, which introduces a premature stop codon at amino acid 337. Accordingly, CSB-PGBD3 is predicted to be absent from the CS1AN-sv cell line, and our anti-CSB immunoprecipitation experiments agree with this prediction (Figure S1).

Stable cell lines expressing CSB were generated by infecting CS1AN-sv cells with CSB-expressing lentivirus (pLenti-PGK-Neo, Addgene) [8]. Stable cell lines expressing CSB or harboring the empty vector were selected with 600 µg/ml G418. CSBΔN1 was expressed from the pSVL vector [8]. CS1AN-sv cells stably expressing CSBΔN1 were generated by cotransfection with pLenti-PGK-neo. After selection with 600 µg/ml G418, single colonies were cloned [8]. CSB^R670W^ was expressed from MSCV-Puro. The stable cell line expressing CSB^R670W^ was generated by transfection and selecting with 250 ng/ml puromycin [35].

### shRNA knockdown

Mission shRNA targeting c-Jun (TRCN0000010366, Sigma) was used to decrease c-Jun protein levels. A non-targeting shRNA (SHC002, Sigma) was used as a negative control. Virus was produced by cotransfecting a 10 cm plate of ∼90% confluent 293T cells with third generation lentivirus packaging plasmids (pMGLg/pRRE, pRSV-REV, and pMD2.G/VSV). A total of 20 µg of plasmid was transfected, with the individual plasmids at an equal molar ratio. The culture medium was changed 24 hours post-transfection, and virus-containing medium was collected 24 hours later. Medium from one plate of virus-producing cells was distributed to six 10 cm dishes of target cells: CS1AN-sv/CSB. The confluence of the target cells at the time of infection was approximately 20%. Infected cells were harvested 36–48 hours post-infection for RNA preparation and western blot analysis.

### Gene expression analysis

Total RNA was prepared using TRIzol (Invitrogen). AMV reverse transcriptase and random primers were used for first strand cDNA synthesis (Roche). cDNA was analyzed by real-time PCR using a MyiQ thermal cycler and SYBR green (BioRad). Expression was first normalized against β-actin and fold over vector-only control was then calculated using ΔΔCt method [57]. Primers used for RT-qPCR are listed in Table S6. ChIP-qPCR assays were performed as previously described [12]. Primers used for ChIP-qPCR are listed in Table S5.

### ChIP and ChIP-western analysis

To increase ChIP efficiency we removed soluble CSB before cross-linking DNA and proteins [8], [35], [58]. Cells were collected in Buffer B (150 mM NaCl, 0.5 mM MgCl_2_, 20 mM HEPES pH 7.8, 10% Glycerol, 0.5% Triton X-100) and soluble CSB was separated from chromatin by centrifugation at 15,000 RPM for 5 min at 4°C. The resulting pellets were resuspended in Buffer B and fixed with 1% formaldehyde for 10 min at room temperature. Cross-linked cells were sonicated at 40% amplitude (30 sec on, 90 sec off, for 24 min total) using the Branson 101-135-126 Sonifier. Chromatin IP (ChIP) was performed using a monoclonal anti-CSB antibody (1B1) that recognizes the N-terminal 507 amino acids of CSB [35], [39] or an anti-c-Jun antibody (Santa Cruz, sc-1694). ChIP samples were reverse cross-linked in SDS sample buffer for subsequent western blot analyses [20]. Antibodies used for western blot analysis are rabbit anti-CSB antibodies (kindly provided by Dr. Alan Weiner, U. Washington) [35], c-Jun (Santa Cruz, sc-1694) and GAPDH (Millipore, MAB374).

### ChIP-seq and data analysis

10 ng of ChIPed DNA was used to prepare libraries for deep sequencing using the multiplexed ChIP-Seq sample preparation protocol described on the website of the Next-Generation Sequencing Core, Perelman School of Medicine, University of Pennsylvania (http://ngsc.med.upenn.edu/). The Next-Generation Sequencing Core at the University of Pennsylvania performed DNA sequencing using Illumina hiSeq2000 sequencers for single-end sequencing with a read length of 50 bps. The resulting sequencing reads were mapped to the human genome (HG19 assembly) using Bowtie version 0.12.7. Peaks were identified using HOMER version 4.1 (Hypergeometric Optimization of Motif EnRichment) with a default option (FDR = 0.001 and Poisson p-value cutoff = 0.0001) on ChIPed samples against matching input DNA samples. Raw and processed files (GSE50171) have been deposited at the Gene Expression Omnibus (GEO) repository. (http://www.ncbi.nlm.nih.gov/geo/query/acc.cgi?token=vfmfryagmygcony&acc=GSE50171).

To classify a peak as unique or common, we determined the intensities (rpm) of the CSB and CSBΔN1 signals within a 200 bp region around a peak center. If the difference between signal intensities was 4-fold or greater and the p-value for that difference was ≤0.0001, the peak was classified as unique. The remaining signals were classified as common. CSB and CSBΔN1 peaks were classified as follows: (1) promoter (from −1 kb to the transcription start site), (2) TTS (from the transcription termination site to +1 kb), (3) 5′ UTR, (4) 3′ UTR, (5) exon, (6) intron, and (7) intergenic (the remainder). The source of gene annotation was UCSC RefGene. The CEAS package [48] computes p-values using one-sided binomial test. To compute p-values for comparisons between CSB and CSBΔN1, we considered a null model in which both classes of peaks form a single population. m and n are the total peak numbers of those classes, and m′ and n′ are the number of peaks in a specific annotation category, and a combined frequency f equal to (m′+n′)/(m+n). p-values for comparisons between CSB and CSBΔN1 are equal to the product of the two p-values from the one-sided binomial test for n, n′ and f, as well as for m, m′ and f.

The Genomic Regions Enrichment of Annotations Tool (GREAT, version 2.0.2) was used for pathway analysis of CSB occupancy sites, using the “MSigDB pathways' category” [59]. The assignment of peaks to genes was made using the following parameters: proximal 5 kb upstream, 1 kb downstream, plus distal up to 1000 kb.

### Comparison of the CSB ChIP-seq data to published CSB-PGBD3 ChIP-seq data

To compare the genomic occupancy of CSB to CSB-PGBD3, we downloaded the ChIP-seq data for CSB-PGBD3 and the matching input from the GEO repository (GSE37919) [39]. The CSB-PGBD3 peaks were called against input using HOMER, and we identified 1,590 peaks. Using binary peak calling (+/−100 bp), we identified 165 peaks as common to CSB and CSB-PGBD3, which represented 1% total CSB and 10% total CSB-PGBD3 peaks (Tables 3 and S3). The hypergeometric p-value was calculated for the AP-1 motif against total CSB as well as CSB-PGBD3 peaks (Table 3). The ontology of the nearby genes was obtained using GREAT (Table 4).

### Co-immunoprecipitation assays

CS1AN cells expressing CSB were lysed in Buffer B60 (20 mM HEPES (pH 7.9), 60 mM NaCl, 0.5 mM MgCl_2_, 1 mM DTT, 0.5% triton X-100 and 20% glycerol) with protease inhibitors (0.5 mM PMSF, 10 µM E64 and 3 µM pepstatin A) and centrifuged at 15,000 rpm for 5 min at 4°C. The supernatant was used for co-immunoprecipitation assays. CSB-containing complexes were recovered using the monoclonal anti-CSB antibody 1B1 (1 hour at 4°C) and protein G agarose beads (30 min at 4°C). The resulting immunocomplexes were washed four times in buffer B60, and the immunoprecipitated proteins were eluted with Laemmli buffer. Beads-only control immunoprecipitations were conducted in parallel.

### Micrococcal nuclease digestion and mononucleosomal DNA purification

Formaldehyde-fixed nuclei were isolated from each cell line as described previously [58], [60]. Nuclei (A_260_ = 500) were incubated with MNase (final concentration 25 U/ml) at 37°C for 10 min and reverse cross-linked at 65°C for 16 hours. After phenol-chloroform extraction, digested DNA was resolved on a 1.2% agarose gel in 1×TAE at 100 V for 3.5 hr. Mononucleosome-size DNA fragments were purified from gel slices and subjected to qPCR analysis (Figure S6). Primers used in MNase-qPCR assays are listed in Table S7.

### CSB peak classification according to chromatin features

CSB, top CSB (rpm greater than 1) and CSBΔN1 peaks were classified using the epigenomic information derived from NHLF (normal human lung fibroblast) cells, via different chromatin features using chromHMM [34], [61]. The region annotation used here is the result of unsupervised learning, finding an HMM with 15 states that minimizes the entropy of observed histone modifications, and afterwards interpreted using prior biological knowledge. Peaks were assigned to regions according to the location of the peak centers. p-values were calculated using a one-sided binomial test.

### Comparisons of CSB ChIP-seq data with transcription profiling data

To compare the CSB ChIP-seq data with the microarray data, we used the lists of up- and down-regulated genes generated by Newman et al. (2006) and Bailey et al. (2012) [28], [29]. Newman et al. identified CSB-responsive genes in hTERT immortalized CS1AN cells. Bailey et al. (2012) identified CSB-responsive, PGBD3-responsive, and CSB+PGBD3-responsive genes in USS1KO cells. We determined the number of genes whose body or promoter regions overlapped with CSB peaks. p-values were calculated after randomly generating 17,779 peaks (the same number of CSB ChIP-seq peaks). Hypergeometric p-values were calculated against the total number of CSB peaks for the c-Jun/Ap-1 motifs.

## Supporting Information

Figure S1 CSB-PGBD3 is not expressed in CS1AN-sv cells reconstituted with CSB. Whole-cell lysates from 293T cells, CS1AN-sv cells, and CS1AN-sv cells reconstituted with CSB were subjected to immunoprecipitation using the monoclonal anti-CSB N-terminal antibody 1B1. The immunoprecipitated material, along with the input lysates, was resolved in a 7% Tris-Acetate gel (NuPAGE) and the western blot was probed with the polyclonal anti-CSB N-terminal antibody. The monoclonal antibody immunoprecipitated both CSB and CSB-PGBD3 from 293T cells, but this antibody only immunoprecipitated CSB from CS1AN-sv cells reconstituted with CSB. Nothing was immunoprecipitated from CS1AN-sv cells. The band marked with an asterisk, present in lanes 1, 2 and 3, is of unknown identity; however, this protein could not be immunoprecipitated with 1B1 and is, therefore, likely cross-reacting with the polyclonal antibody.
(TIFF)

Figure S2 Genomic distributions of CSB and CSBΔN1. The genome was divided into seven categories defined by the UCSC RefSeq gene annotation. The genomic distributions of CSB and CSBΔN1 were determined using the CEAS package and are presented as pie charts [32]. The table includes p-values for these distributions.
(TIFF)

Figure S3 CSB and CSBΔN1 occupancy positions relative to transcription start sites. The number of CSB and CSBΔN1 binding sites were plotted as a function of distance from transcription start sites (TSS) using a bin size of 500 bp.
(TIFF)

Figure S4 Genomic distributions of peaks common or unique to CSB and CSBΔN1. Distributions were determined using the CEAS package and are presented as pie charts. The table includes p-values for these distributions.
(TIFF)

Figure S5 Genomic distribution of CSB peaks containing TPA-response element. Distributions were determined using the CEAS package and are presented as a pie chart. The table includes p-values for these distributions.
(TIFF)

Figure S6 Preparation of mononucleosomal DNA for MNase sensitivity assays. (A) After limited MNase digestion of formaldehyde cross-linked nuclei, the cross-links were reversed, and the DNA was purified and resolved in an agarose gel. (B) Mononucleosomal DNA purified from (A) and used in the qPCR assays.
(PDF)

Figure S7 MNase-qPCR analysis of the WDR74 promoter region. Upper panel from top to bottom: screen shots from the UCSC genome browser (GRCh37/hg19 Assembly) showing the RefSeq gene and direction of transcription (arrow head), the position of CSB occupancy (CSB ChIP), the position of CSBΔN1 occupancy (CSBΔN1 ChIP), ENCODE MNase-seq data obtained from K562 cells (MNase), and the amplicons used in the MNase-qPCR assays (Amplicon). The chromosome coordinates shown are chr11:62,608,860-62,609,234. Lower panel contains bar graphs showing results from the MNase-qPCR assays. The primers used in the MNase-qPCR assays are listed in Table S7. Shown are means +/− SEM.
(TIFF)

Table S1 Genomic annotation of peaks common to CSB and CSBΔN1.
(XLSX)

Table S2 Genomic annotation of peaks unique to CSB.
(XLSX)

Table S3 Common sites of CSB and CSB-PGBD3 occupancy.
(XLSX)

Table S4 Lists of genes correlated in Tables 5 and 6.
(XLSX)

Table S5 Primers used in ChIP-qPCR assays.
(DOCX)

Table S6 Primers used in RT-qPCR assays.
(DOCX)

Table S7 Primers used in MNase-qPCR assays.
(DOCX)
