# Supplementary figures and images for: The Sequence-Specific Transcription Factor c-Jun Targets Cockayne Syndrome Protein B to Regulate Transcription and Chromatin Structure
Source: PLoS Genet. 2014 Apr 17;10(4):e1004284. doi: 10.1371/journal.pgen.1004284 (PMC3990521; doi:10.1371/journal.pgen.1004284)

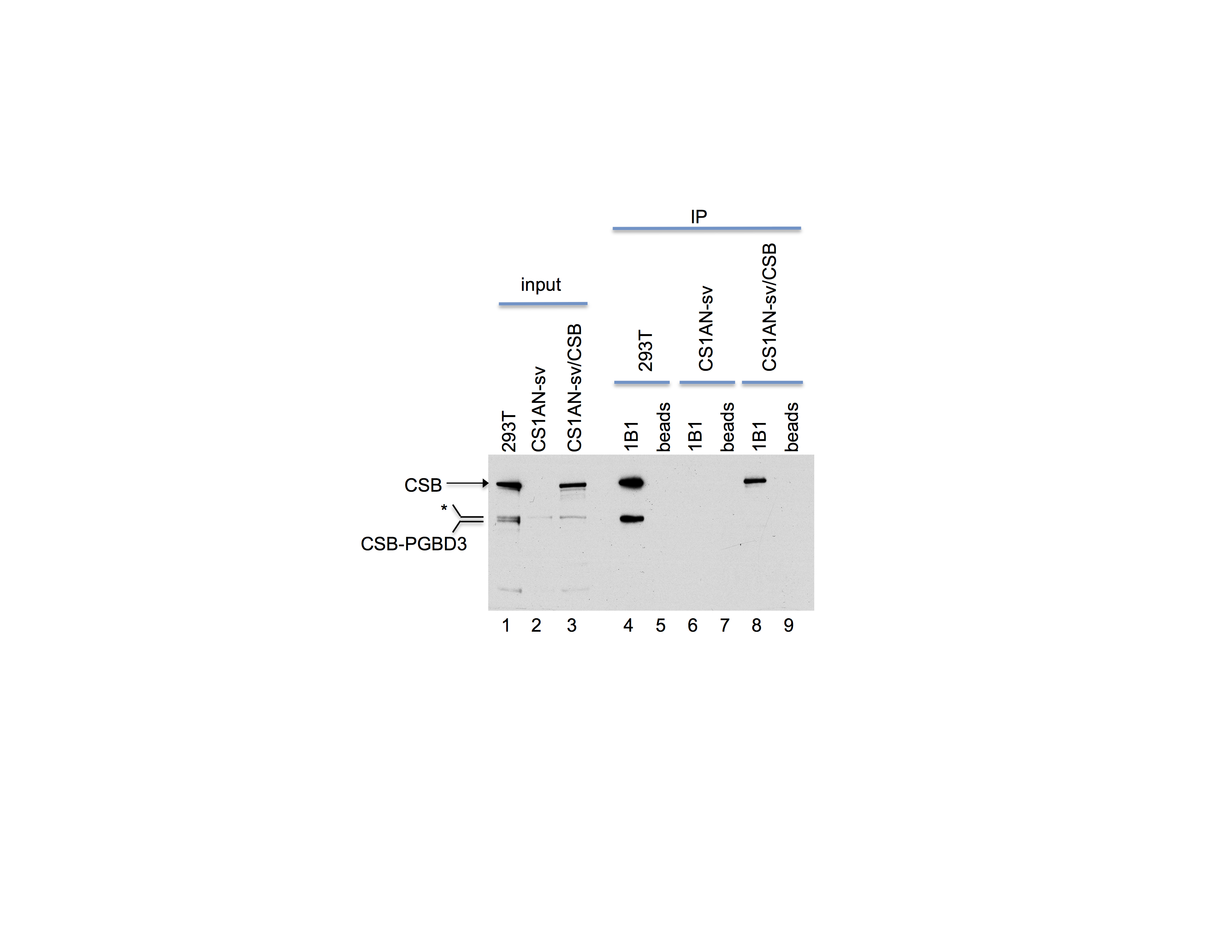

Supplement: Figure S1 — CSB-PGBD3 is not expressed in CS1AN-sv cells reconstituted with CSB. Whole-cell lysates from 293T cells, CS1AN-sv cells, and CS1AN-sv cells reconstituted with CSB were subjected to immunoprecipitation using the monoclonal anti-CSB N-terminal antibody 1B1. The immunoprecipitated material, along with the input lysates, was resolved in a 7% Tris-Acetate gel (NuPAGE) and the western blot was probed with the polyclonal anti-CSB N-terminal antibody. The monoclonal antibody immunoprecipitated both CSB and CSB-PGBD3 from 293T cells, but this antibody only immunoprecipitated CSB from CS1AN-sv cells reconstituted with CSB. Nothing was immunoprecipitated from CS1AN-sv cells. The band marked with an asterisk, present in lanes 1, 2 and 3, is of unknown identity; however, this protein could not be immunoprecipitated with 1B1 and is, therefore, likely cross-reacting with the polyclonal antibody. (TIFF) [file pgen.1004284.s001.tif]

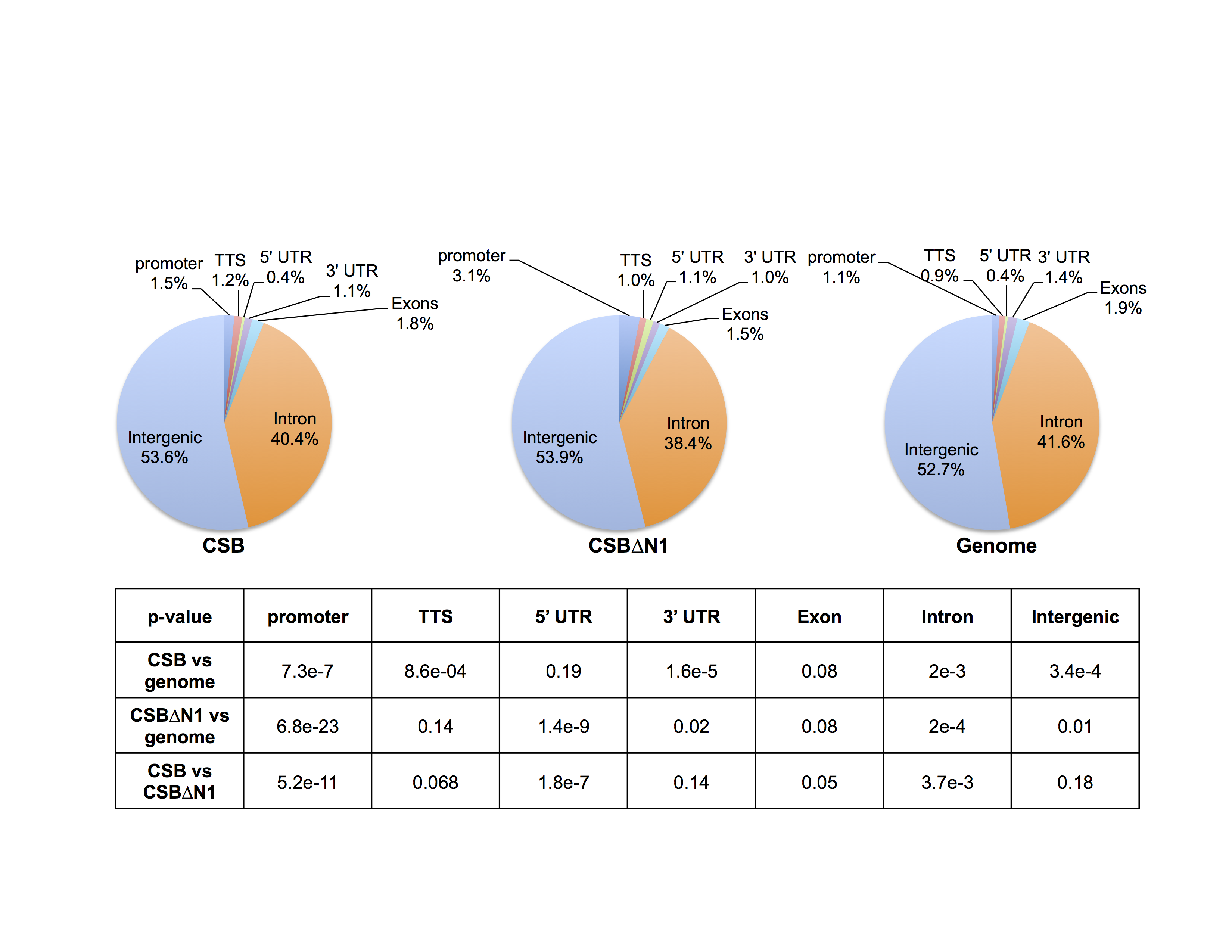

Supplement: Figure S2 — Genomic distributions of CSB and CSBΔN1. The genome was divided into seven categories defined by the UCSC RefSeq gene annotation. The genomic distributions of CSB and CSBΔN1 were determined using the CEAS package and are presented as pie charts [32]. The table includes p-values for these distributions. (TIFF) [file pgen.1004284.s002.tif]

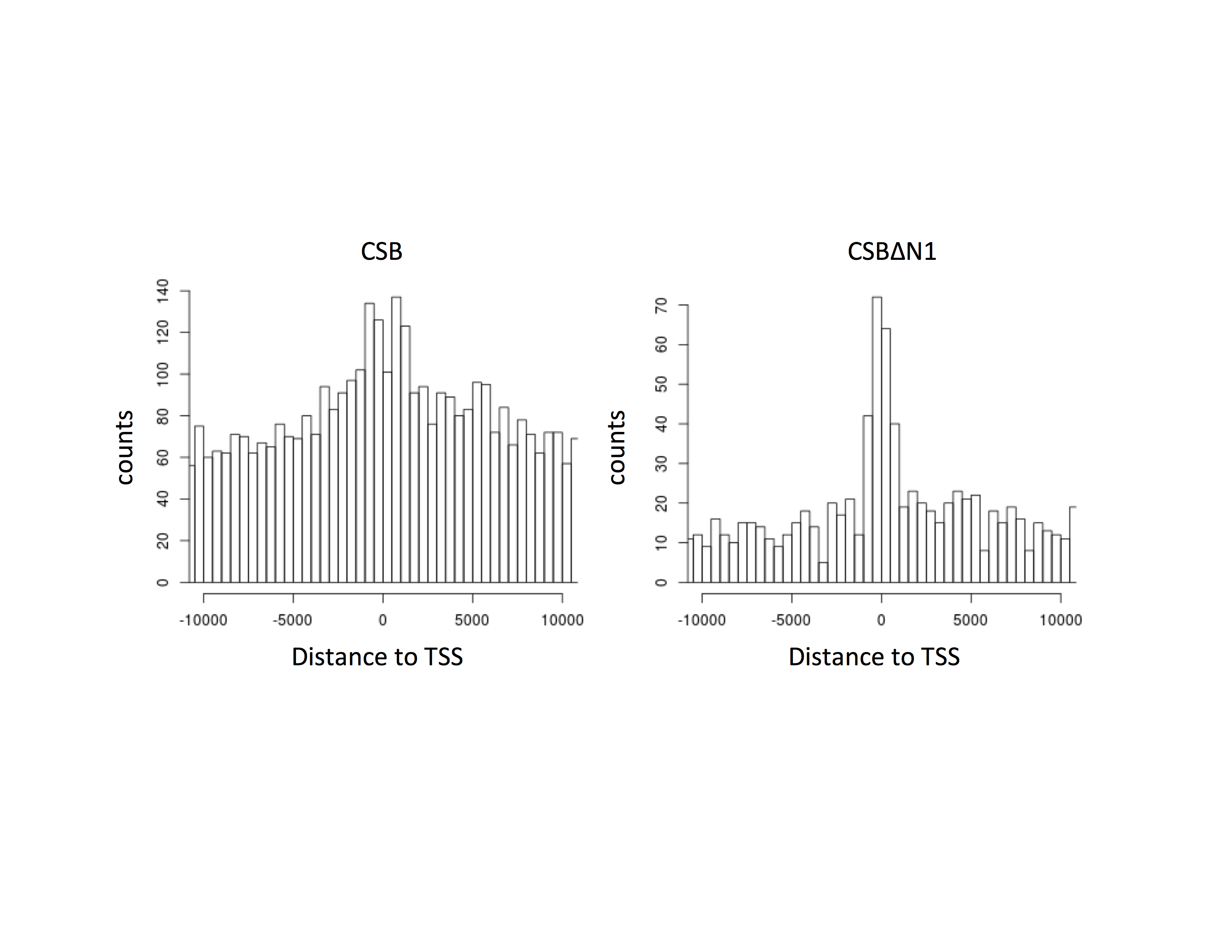

Supplement: Figure S3 — CSB and CSBΔN1 occupancy positions relative to transcription start sites. The number of CSB and CSBΔN1 binding sites were plotted as a function of distance from transcription start sites (TSS) using a bin size of 500 bp. (TIFF) [file pgen.1004284.s003.tif]

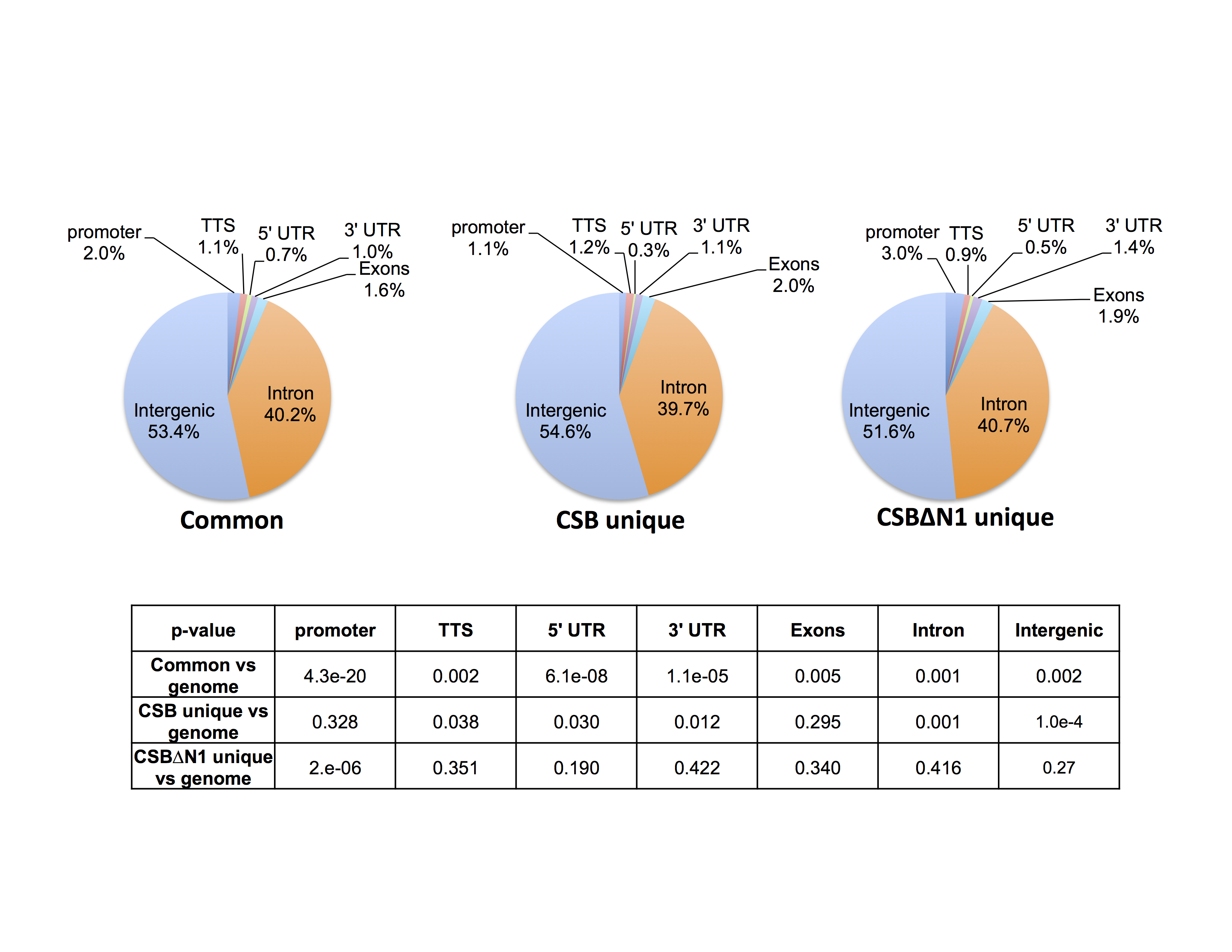

Supplement: Figure S4 — Genomic distributions of peaks common or unique to CSB and CSBΔN1. Distributions were determined using the CEAS package and are presented as pie charts. The table includes p-values for these distributions. (TIFF) [file pgen.1004284.s004.tif]

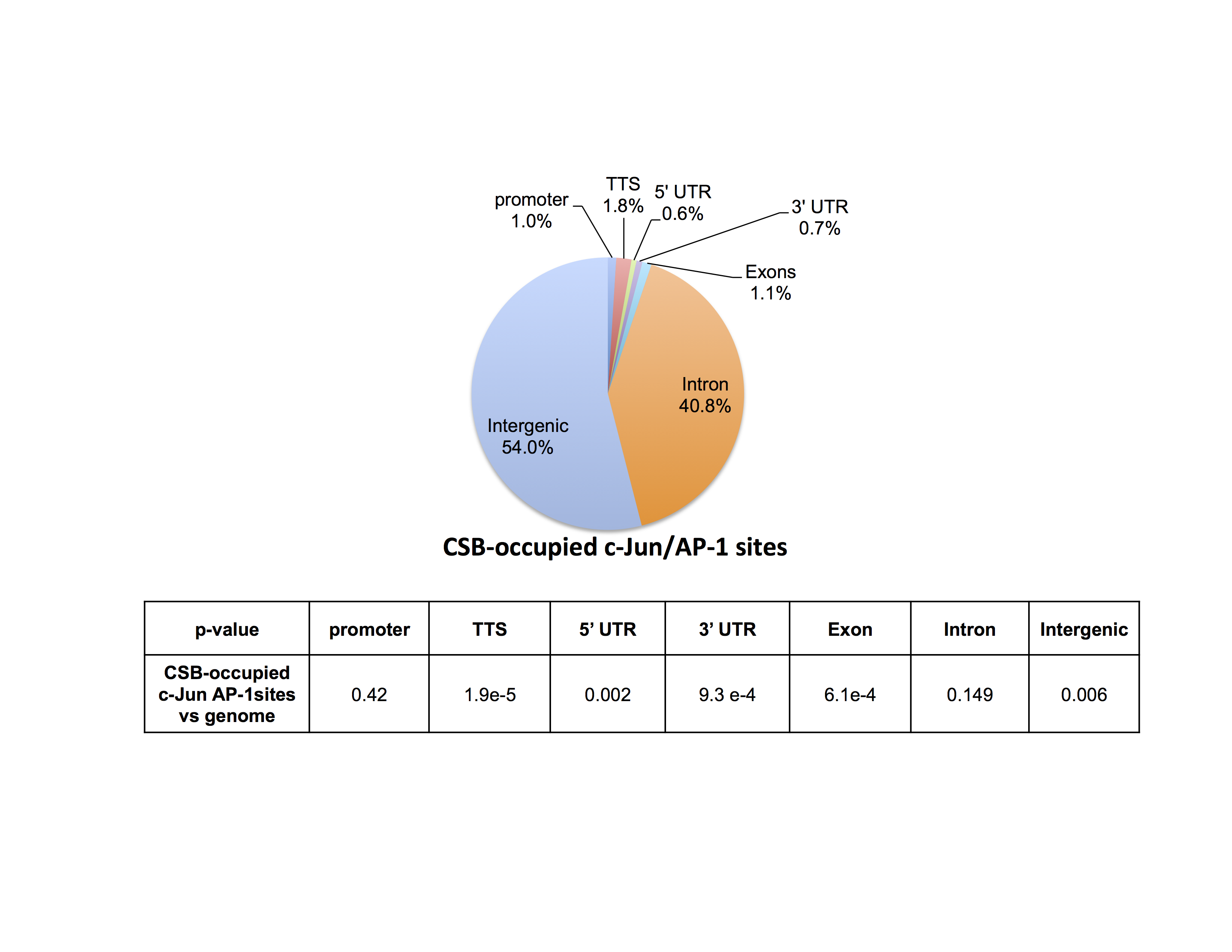

Supplement: Figure S5 — Genomic distribution of CSB peaks containing TPA-response element. Distributions were determined using the CEAS package and are presented as a pie chart. The table includes p-values for these distributions. (TIFF) [file pgen.1004284.s005.tif]

### A. MNase digested DNA

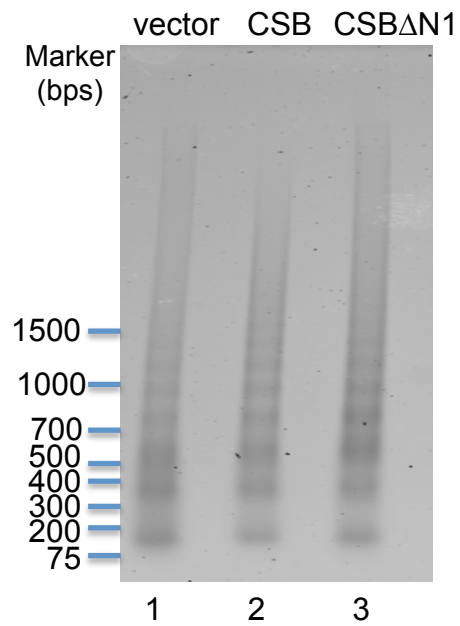

### B. Mononucleosomal DNA after gel purification

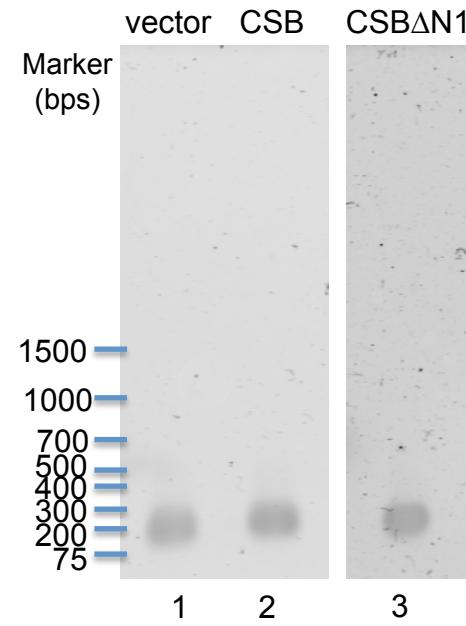

Supplement: Figure S6 — Preparation of mononucleosomal DNA for MNase sensitivity assays. (A) After limited MNase digestion of formaldehyde cross-linked nuclei, the cross-links were reversed, and the DNA was purified and resolved in an agarose gel. (B) Mononucleosomal DNA purified from (A) and used in the qPCR assays. (PDF) [file pgen.1004284.s006.pdf]

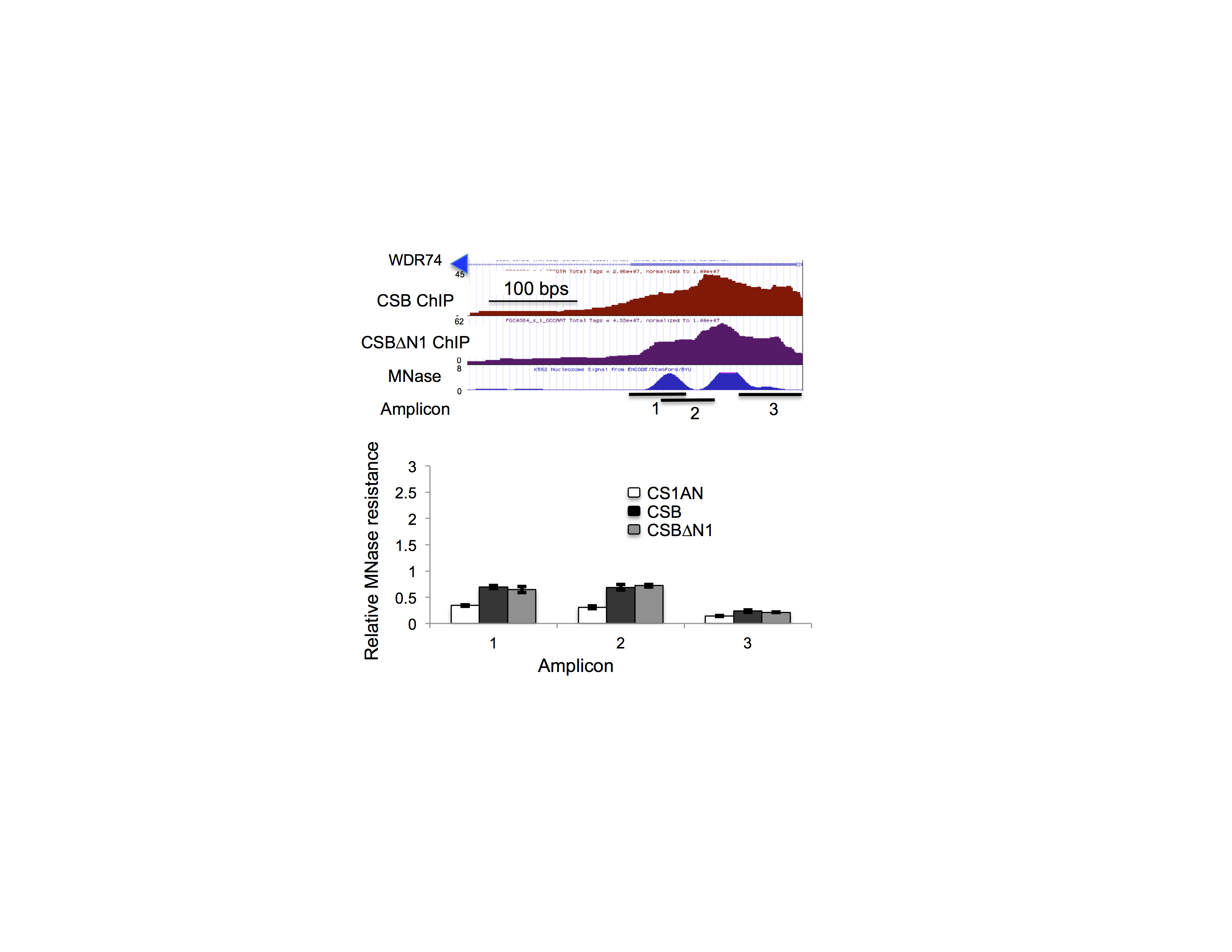

Supplement: Figure S7 — MNase-qPCR analysis of the WDR74 promoter region. Upper panel from top to bottom: screen shots from the UCSC genome browser (GRCh37/hg19 Assembly) showing the RefSeq gene and direction of transcription (arrow head), the position of CSB occupancy (CSB ChIP), the position of CSBΔN1 occupancy (CSBΔN1 ChIP), ENCODE MNase-seq data obtained from K562 cells (MNase), and the amplicons used in the MNase-qPCR assays (Amplicon). The chromosome coordinates shown are chr11:62,608,860-62,609,234. Lower panel contains bar graphs showing results from the MNase-qPCR assays. The primers used in the MNase-qPCR assays are listed in Table S7. Shown are means +/− SEM. (TIFF) [file pgen.1004284.s007.tif]
