# Supplementary material for: The Sequence-Specific Transcription Factor c-Jun Targets Cockayne Syndrome Protein B to Regulate Transcription and Chromatin Structure
Source: PLoS Genet. 2014 Apr 17;10(4):e1004284. doi: 10.1371/journal.pgen.1004284 (PMC3990521; doi:10.1371/journal.pgen.1004284)
Supplement: Table S5 — Primers used in ChIP-qPCR assays. (DOCX) [file pgen.1004284.s012.docx]

**Table S5. Primers used in ChIP-qPCR**

| Name of primer set | Primer name | Sequence (5’ to 3’) |
| --- | --- | --- |
| HES1 | hHES1 -299-Fw | AAG TTT CAC ACG AGC CGT TC |
|  | hHES1 -200-Rv | GAG AGG TAG ACG GGG GAT TC |
| chr1-1 | chr1_236260696 | CAG GTG ACC AGG GGA ATA GA |
|  | chr1_236260779 | TCG TTC TCC TCA GCC CTA GT |
| chr2-2 | chr2_180325437 | CCA GTC CTG TGG TCC TGA CT |
|  | chr2_180325517 | TTG TTT GGC AAT GAA ATC GT |
| chr4-1 | chr4_72978371 | GCC TGA AGC ATG AAA AGA CA |
|  | chr4_72978473 | CAC ATG ACT CAC AGA TGT CAC AAT A |
| chr7-1 | chr7_200,695 | TCA CGT GTT GTG GGA GTG AT |
|  | chr7_2001793 | TAT CTC CCA CCG TGT CCT TC |
| chr17-1 | CHR17-1for | TGC GAA TTT CCA TGG GTT AT |
|  | CHR17-1Rev | GTC AAC ATT GGC TGA AGC TG |
| chr20-1 | chr20ChIP_47895161F | TTC AGG AAG CCA TTC GTT CT |
|  | chr20ChIP_47895291R | TGG AGT TTC CAC TCA CCA CA |
| chrX-1 | chrX-1for | GTC TTG ACC ACA TGT GAC TGG |
|  | chrX-1Rev | AGC TGG TCT AAG CCG ATC AA |
