# Supplementary material for: The Sequence-Specific Transcription Factor c-Jun Targets Cockayne Syndrome Protein B to Regulate Transcription and Chromatin Structure
Source: PLoS Genet. 2014 Apr 17;10(4):e1004284. doi: 10.1371/journal.pgen.1004284 (PMC3990521; doi:10.1371/journal.pgen.1004284)
Supplement: Table S6 — Primers used in RT-qPCR assays. (DOCX) [file pgen.1004284.s013.docx]

**Table S6. Primers used in RT-qPCR**

| Name of primer set | Primer name | Sequence (5’ to 3’) |
| --- | --- | --- |
| ACTB | hACTB_for | CGC TCG TCG TCG ACA ACG |
|  | hACTB_rev | CTC TGG GCC TCG TCG CC |
| DPP9 | DPP9F | TCA ATA ACA GCG ACC TGT GG |
|  | DPP9R | GCG GTC GAA CTC TTC CTG TA |
| ZNFX1-NC1 | hZNFX1NC1-F1 | AAG CCA CGT GCA GAC ATC TA |
|  | hZNFX1NC1-R1 | CTA CTT CCA ACA CCC GCA TT |
| MAD1L1 | MAD1L1F | TGT CAG CAG AAC TTG GAT GC |
|  | MAD1L1R | CAC TGC AGT TCC GAG ATC CT |
| MCPH1 | MCPH1F | GAT CCC GCC GTC TGT CAT |
|  | MCPH1R | CCC CCA TAT CCA CAA GCT G |
| NPFFR2 | NPFFR2_F | TCG CAG CTT CAG TCT TTA CG |
|  | NPFFR2_R | GAT GGC TAG GAC CCA GAT GA |
| PRMT5 | PRMT5F | ATG CAC CAG AGG CTC ATC TT |
|  | PRMT5R | TTA GGT GGA GGA CGG TTC TG |
| SACM1L | SACM1L181F | GAA GGA AGG AGG TGG TTG TG |
|  | SACM1L320R | TCT GTG GAC ACA CGG TCA AT |
| WDR74 | WDR74F | GCT CTG AGG AAC CTG TGT TCA |
|  | WDR74R | AGG GTC ATG GCT GTT AGT GG |
| ZNF385B | ZNF385B_F | CCG AAG GCA TAA AGA TCG AG |
|  | ZNF385B_R | GGA ATG CAA GTT TTG CTG CT |
| MSANTD3 | MSANTD3F | TTC TGA GCC AGT GGG AAC TC |
|  | MSANTD3R | AGC AGG ATG CTC TTT TCC AA |
| ZNF507 | ZNF507_12F | CCA GTG CAA GCA GTG TGA AG |
|  | ZNF507_12R | TGG ACA CAT TTT CCA TCA GC |

|  |  |  |  |
| --- | --- | --- | --- |
|  |  |  |  |
|  |  |  |  |
|  |  |  |  |
|  |  |  |  |
|  |  |  |  |
|  |  |  |  |
|  |  |  |  |
|  |  |  |  |
|  |  |  |  |
|  |  |  |  |
