# Supplementary material for: The Sequence-Specific Transcription Factor c-Jun Targets Cockayne Syndrome Protein B to Regulate Transcription and Chromatin Structure
Source: PLoS Genet. 2014 Apr 17;10(4):e1004284. doi: 10.1371/journal.pgen.1004284 (PMC3990521; doi:10.1371/journal.pgen.1004284)
Supplement: Table S7 — Primers used in MNase-qPCR assays. (DOCX) [file pgen.1004284.s014.docx]

Table S7. Primers used in MNase-qPCR

| Name of primer set | Primer name | Sequence (5’ to 3’) |
| --- | --- | --- |
| chr17-1 | chr17-1for | TGC GAA TTT CCA TGG GTT AT |
|  | chr17-1Rev | GTC AAC ATT GGC TGA AGC TG |
| chrX-1 | chrX-1for | GTC TTG ACC ACA TGT GAC TGG |
|  | chrX-1Rev | AGC TGG TCT AAG CCG ATC AA |
| ZNFX1-NC1, #1 | chr20ChIP_47895161F | TTC AGG AAG CCA TTC GTT CT |
|  | chr20ChIP_47895291R | TGG AGT TTC CAC TCA CCA CA |
| ZNFX1-NC1, #2 | ZFAS-119F | GCTATTGTCCTGCCCGTAAG |
|  | NFAS196R | GACCAGAGGTCTCCAACGAA |
| ZNFX1-NC1, #3 | NFAS-185F | AGA CCT CTG GTC CTC CCT TC |
|  | NFAS-280R | CCT ATT TGG GGT CAG AGA CG |
| ZNFX1-NC1, #4 | ZFAS-318F | AAA AGC CAC TGC CTT CTG C |
|  | NFAS-400R | GTCGATCTGATGGGGAAAAA |
| ZNFX1-NC1, #5 | NFAS-381F | TTTTTCCCCATCAGATCGAC |
|  | NFAS-479R | ACGGTCAGAAACGAACAAGG |
| PRMT5,  #1 | PRMT5L_36F | GTCGCCTTAACAACCAGAGC |
|  | PRMT5L_107R | ATTGGCAGGAAAAGCCACT |
| PRMT5,  #2 | PRMT5L_89F | AGTGGCTTTTCCTGCCAAT |
|  | PRMT5L_160R | ACCAGACCCTGAGATTGGTG |
| PRMT5,  #3 | PRMT5L_134F | ATGGATCCACCAATCTCAGG |
|  | PRMT5L_213R | AACGGCGTTCTTCAGATAGAC |
| PRMT5,  #4 | PRMT5L_143F | CCAATCTCAGGGTCTGGTTC |
|  | PRMT5L_220R | GTCTCTCAACGGCGTTCTTC |
| PRMT5,  #5 | PRMT5R_50F | GCTGCGTGAGCATTTTGTAA |
|  | PRMT5R_128R | TCTGATAATGAAAGCGTGAAGA |
| PRMT5,  #6 | PRMT5R_124F | TCAGATCCTGAGTCGGTTGG |
|  | PRMT5R_184R | GGGTAGTTACGGGAAAAGCTG |
| PRMT5,  #7 | PRMT5R_182F | CCCTCAAAAGTGTTTTTCTTGTG |
|  | PRMT5R_255R | CACCAGCCATTAACCCAGTT |
| WDR74  #1 | WDR74_32F | CCCTAACTGATCGAAATCTTCC |
|  | WDR74_97R | WDRAACGGTGCACTCTCCCTTC |
| WDR74  #2 | WDR74_65F | GTTGTTCTCTCCCCGAAGG |
|  | WDR74_129R | ACGCATCGACCTGGTATTG |
| WDR74  #3 | WDR74_88F | GTGCACCGTTCCTGGAAGTA |
|  | WDR74_162R | GGAGATGGAATAGGAGCTTGC |
| MAD1L1 #1 | MAD1L1_3F | CCC CCA TAC CAT TCT CAT CT |
|  | MAD1L1_72R | GGG AAA CCA TCC CTG TGA TT |
| MAD1L1 #2 | MAD1L1_20F | TCT ATT GTG GGA AGG ACA CG |
|  | MAD1L1_95R | TCA CGA CCA CGA GAA TGG TA |
| MAD1L1 #3 | MAD1L1_76F | TAC CAT TCT CGT GGT CGT GA |
|  | MAD1L1_154R | AGA ATG AGA GCC AAG CCA AA |
| MAD1L1 #4 | MAD1L1_109F | GAT CTG ATT TTA TAA GGG GTT TCC |
|  | MAD1L1_190R | GGC AAA AGG CAC ATC TTA CA |
| MAD1L1 #5 | MAD1L1_132F | CCC TTT GGC TTG GCT CTC |
|  | MAD1L1_208R | CTC ACA ATC ATG GCA GCA G |
| MAD1L1 #6 | MAD1L1_312F | TCC AAA GGA TCA TAT ATA ACA GAA AA |
|  | MAD1L1_400R | TTC GTT CTT TCA TCA GGT ATT TTG |
